# Evaluation of food waste treatment techniques using aczel alsina based MAGDM model in the q-rung orthopair fuzzy soft structure

**DOI:** 10.1038/s41598-025-09082-z

**Published:** 2025-07-18

**Authors:** Rana Muhammad Zulqarnain, Hongwei Wang, Usman Zulfiqar, Rifaqat Ali, Imran Siddique, Abdullatif Saleh Ghallab, Hafiz Shahzar Riaz Khan Tareen, Sohaib Abdal

**Affiliations:** 1 School of Economics and Management, Tongji University, No. 1239 Siping Road, Yangpu District, Shanghai, China; 2 Department of Economics, Western Caspian University, Baku, Azerbaijan; 3 Department of Business Administration, Lahore Leads University, Lahore, Pakistan; 4 Department of Mathematics, Applied College in Mohayil Asir, King Khalid University, Abha, Saudi Arabia; 5 Department of Mathematics, University of Sargodha, Sargodha, 40100 Pakistan; 6 Mathematics in Applied Sciences and Engineering Research Group, Scientific Research Center, Al-Ayen University, Nasiriyah, 64001 Iraq; 7 Faculty of Computing and Information Technology, University of Science and Technology, Sana’a, Yemen; 8 Department of Statistics, Bahauddin Zakariya University, Multan, 60000 Pakistan; 9 Department of Mathematics, Saveetha School of Engineering, SIMATS Thandalam, Chennai, Tamilnadu 602105 India

**Keywords:** q-rung orthopair fuzzy soft set, Aczel Alsina aggregation operators, Multi-attribute group decision-making, Food waste treatment techniques

## Abstract

Food waste is a major obstacle in managing inequality, optimizing living conditions, and promoting prosperity, specifically among the world’s most starving economies. Its influences stretch to preventing food supply; it alters financial maturation, complicates environmental issues decomposition, and incorporates raised food operating expenses. Monitoring food waste is implicitly challenging due to confusion arising from its authenticity, extent, geographic location, and schedule; all factors prevent decision-making procedures. This research proposes Aczel–Alsina operational laws to solve the obstacles and intrinsic uncertainty in a q-rung orthopair fuzzy soft sets (q-ROFSS) structure. Also, two novel Aczel–Alsina aggregation operators (AOs) such as q-rung orthopair fuzzy soft aczel–alsina weighted average (q-ROFSAAWA) and q-rung orthopair fuzzy soft aczel–alsina weighted geometric (q-ROFSAAWG) operators are developed with their desirable properties. These operators encourage more accurate and sustainable consolidation of unsure data in multi-attribute group decision-making (MAGDM) mechanisms. A real-life example highlights the proposed method’s feasibility and efficacy in identifying the most optimal food waste treatment technologies (FWTT). The comparative study confirms this methodology’s validity, exactitude, and feasibility, clarifying its better accuracy and feasibility as compared to other methods. The outcomes demonstrate that the most effective technique for facilitating food waste treatment in the FWM is incineration.

## Introduction and literature review

Food waste not only stresses the socioeconomic growth of a country but also states the improper utilization of natural resources such as energy, water, and land. Some of the most significant elements of food waste management are insufficient climate change management, excess manufacturing, undetermined economies, and improper consumer figures of constituents that affect it. Food waste originates from ordering excessively in grocery stores and from over-preparation of meals. Worldwide food waste seriously impacts the financial system, community, and surroundings. Food that people refrain from eating generates approximately 8–10% of the releases of greenhouse gases all over the world.

The report claims that since 2014, the total number of people severely affected by starvation who do not have necessary food has increased^1^. Wasted food tracks demonstrate qualitative and statistical implications of the decline in the food supply network and food waste. The farming industry started to build up upon preserves to find adequate ground, influencing our surroundings and growing farming production. The decomposition of food in landfills emits methane, a greenhouse gas that is twenty-five times more potent than carbon dioxide. The 2024 indicate^2^ states that Pakistan’s annual food waste is higher than that of several other countries, but it remains substantial. As the proportion of Pakistanis continues to increase, FWM continues to pose severe problems. In ceremonies, restaurants, hotels, family or social meetings, and homes, a lot of food is discarded.

**Fig. 1:**
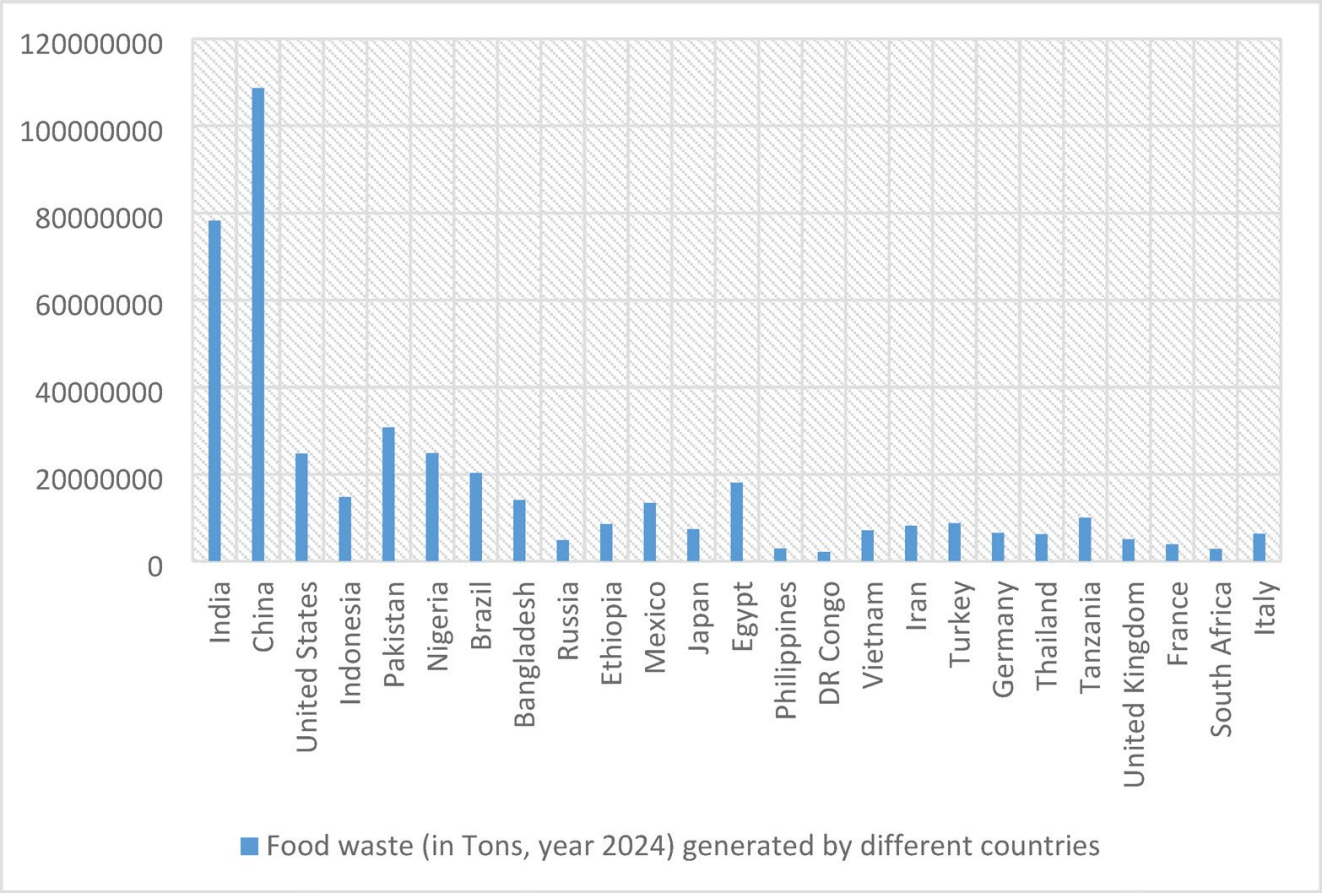
Food waste generated by different countries in 2024.

However, Pakistan’s FWM is a devastating challenge. All the evidence establishes that our dumps, waste bins, and roads interrupt the natural environment. Figure 1 illustrates^3^ the yearly tons of wasted food generated globally.

Also, remember that inefficient FWM eliminates natural resources, violates the welfare of people, and undermines rivers and the oceans. Other than regional, state, and federal government responses, the global rewards of minimizing food wastage and loss depend on FWM. However, it developed multiple decades prior, and the FWTT was only viewed as academically stimulating with little implementation merit. Its main objective is to maintain the environment and space by minimizing the consumption of facilities for dumping and petroleum-derived substances. However, the importance of generating renewable energies and recognizing climate change has recently triggered their benefits to be demonstrated. Food debris is one of the core sources of waste, providing an excellent volume but a minimal reuse and recycling rate. From a sustainability and financial perspective, environmentally friendly FWM uses techniques that reduce the detrimental impact of food waste. The decision to use FWM is complicated and can be examined as a MAGDM challenge and initiated to deal with the FWM, expected alternate FWTT to have distinctive biological, monetary, social, and scientific outcomes about medication, execution, transportation, etc. The most suitable FWTT is a MAGDM problem; numerous factors are considered in the decision-making process.

Numerous investigations have examined different strategies for FWM. Still, because of the growing difficulties of external conditions and the built-in subjective aspects in individual decisions, official analyses of obtainable alternates often demonstrate inconsistency or imperfection. The research offers various investigations on FWM implementing distinct fuzzy structures. Chen et al.^4^ stated a hybrid approach implementing entropy and the Analytic Hierarchy Process (AHP) to examine the protection of food waste. By implementing a fuzzy mathematical approach, Chen et al.^5^ determined metabolic oxidation devices’ financial and environmental advantages for methane extraction from food waste. Choi and Park^6^ discussed anaerobic composting strategies that merge agricultural evaporation with animal wastewater farming.

Irani et al.^7^ proposed a methodical technique to evaluate the main components facilitating productive FWM using fuzzy cognitive maps to locate and examine correlations between administrative variables. Morone et al.^8^ constructed a fuzzy-based structure to encourage sustainable food consumption arrangements. Ali et al.^9^ substantially contributed to regulating food supply chains. To meticulously perform and adhere to retail food waste’s determinants and associated aspects, YaranÖgel et al.^10^ applied a fuzzy best–worst method utilizing Dombi-Bonferroni operators. Genc and Ekici^11^ stated an efficient and exhaustive method for determining the significant variables determining the existence of house food debris, which provides an accurate representation to more accurately recognize the details and dynamics involved in common food waste generation.

Buyuk and Temur^12^ formulated a multi-criteria decision-making (MCDM) technique integrating the AHP in a spherical fuzzy STRUCTURE to assess the most efficient FWTT. Tripathi et al.^13^ laid out a decision-support model for reviewing FWTT, using intuitionistic fuzzy sets (IFS) to solve unclear and imperfections in the decision-making procedure. Rani et al.^14^ stated a study on the selection of an FWTT within a Fermatean fuzzy framework, using Heronian mean AOs with the MEREC-based additive ratio evaluation tool to enhance decision-making stability. Malibari et al.^15^ presented a fuzzy logic-based technique for promoting FWM methods in the Kingdom of Saudi Arabia, presenting an organized system for efficient disposal solutions. A lot of studies, particularly within the context of chaotic dynamics, focused on the evaluation of singular characteristics, discontinuities, and system characteristics^16^. These ideas are required for predicting confusion, vagueness, and different perspectives, corresponding to challenges encountered in MAGDM contexts, in which numerous options are analyzed under unclear configurations^17–21^.

The flexibility of conventional fuzzy sets (FS)^22^ to express unpredictability and ambiguity resulted in their broad application in various fields of science. To boost this capacity to encode convoluted and uncertain data, investigators developed multiple modifications to the traditional FS structure over the years. These modifications have IFS^23^, Pythagorean fuzzy sets (PyFS)^24^, and q-rung orthopair fuzzy sets (q-ROFS)^25^. These mathematical structures normally will not effectively handle details in parametric decision-making systems where alternatives are determined and analyzed. Molodtsov^26^ offered the theory of soft sets (SS), a robust and adaptable conceptual application, as a substitute for those drawbacks. Intuitionistic fuzzy soft sets (IFSS)^27^, Pythagorean fuzzy soft sets (PyFSS)^28^, and q-rung orthopair fuzzy soft sets (q-ROFSS)^29^ constitute a few of the powerful hybrid structures that were developed from this basic structure, presenting more secure mechanisms for decision-making under indeterminacy.

Triangular norms (T-norms or TNs) are essential in many different FS structures and are significant for forming different AOs, which were thoroughly explored in the prevailing study. The idea of T-norms was first proposed by Menger^30^, who noted its significance in analytics. Deschrijver et al.^31^ explored the implementation of T-norms and their dual counterparts, T-conorms (TCNs), from the perspective of IFS, designing upon this fundamental approach. A multitude T-norms are generated gradually that facilitate predictable and efficient consolidation of multiple conceptual frameworks, which increases the potential of fuzzy networks to navigate exacerbated decisions and unpredictability.

Aczel and Alsina^32^ stated a new category of TNs and TCNs, denoted Aczel–Alsina TNs (AA-TNs) and Aczel–Alsina TCNs (AA-TCNs), which can be modified using the value of $0 \le p \le +\infty$. These mechanisms indicate empirical consistency and persistence as the parameters $p$ alter, facilitating adaptation and versatility in simulating unpredictability. The most common uses of the Aczel–Alsina TNs and TCNs are in various fields to determine the possible outcomes of diverse elements in complex decision-making. Senapati et al.^33^ explored the effective use of Aczel–Alsina operations in IFS and designed AOs that used these mathematical operations to handle multi-attribute decision-making (MADM) obstructions. Ali et al.^34^ demonstrated novel Aczel–Alsina-based AOs for intuitionistic hesitant fuzzy sets, presenting efficient techniques for the MADM problem. Shi et al.^35^ described a unique approach for open-vocabulary semantic splitting, which uses major language representations to boost object description. Mahmood et al.^36^ explored the utilization of AA-TNs and AA-TCNs in complicated IFS contexts, proposing a comprehensive selection of AOs focused on overcoming intricate MADM challenges.

Senapati et al.^37^ designed a strategy using Aczel–Alsina power AOs into IFS to find effective and realistic options in transportation-sharing applications. Hussain et al.^38,39^ stated dynamic Aczel–Alsina operations optimized for PyFS and produced multiple AOs built on these techniques to boost MADM algorithms. Senapati et al.^40^ succeeded in this study by constructing various AOs derived from the Aczel–Alsina operations used in the PyFS structure. They proposed a practical MADM approach predicated on these operators. Senapati et al.^41^ demonstrated improved Aczel–Alsina–based average AOs for q-ROFS, depicting their potential within MADM demonstrations. Farid et al.^42^ constructed Aczel–Alsina AOs for q-ROFS, presented their beneficial algebraic characteristics and exploited them to develop an advanced decision-making strategy.

Hamid et al.^43^ explored the algebraic layout and operating concept of q-ROFSS. They invented TOPSIS and VIKOR algorithms to address multi-criteria group decision-making (MCGDM) complications. Chinram et al.^44^ described geometric AOs for q-ROFSS and explored their related mathematical advantages. Riaz and Farid^45^ proposed the VIKOR method in the q-ROFSS structure and used their developed method to solve a multi-criteria decision-making problem in agricultural land. Zulqarnain et al.^46–48^ stated interactive and Einstein operational rules for q-ROFSS and articulated multiple AOs inspired by such novel operations. To demonstrate their significance in convoluted managerial decisions, Sivadas and John^49^ developed the extended similarity and entropy measures in the q-ROFSS structure. Akram et al.^50^ stated four novel q-rung orthopair fuzzy soft Yager-based AOs, described their conceptual characteristics and exploited them to produce a reliable MAGDM framework for clinical diagnosis. Zeb et al.^51^ introduced the algebraic operational laws for Fermatean fuzzy soft sets with their fundamental properties and developed an MCDM model for the symptomatic treatment of COVID-19 disease. Khan et al.^52–55^ developed several aggregation operators in the q-rung orthopair fuzzy hypersoft structure and utilized their established operators in the decision-making models.

The research described here focuses on clarifying the treatment of food waste strategies in the FWM industry. It implements an Aczel–Alsina–based MAGDM mechanism in the q-ROFSS structure to assess the ideal FWTT. This approach strives to help decision-makers maximize the beneficial outcomes of FWM operations. This technique facilitates decision-making by bursting deficiencies in the prevailing studies, organizing insufficient facts, promoting expert perspectives with research findings, and implementing a reference-based assessment strategy that safely eliminates ranked reverse barriers. The main objective of this research is to establish an organized and logical decision-making procedure for FWTT selection with the intention of providing awareness of any prevailing shortcomings in this field.

The strategy tries to give executives a credible and logical resource for choosing and assessing FWTT, which helps lower expenses, promote operational effectiveness, and stimulate profitable business growth. This section explores the inspiration and scientific concerns that constitute this research.

### Motivation and research problems

Food waste management (FWM) is a gradually significant problem in the world that influences nutritional safety, resource optimization, a sustainable environment, and financial prosperity. The complications that involve food waste are complicated; it’s recommended that precise decision-making be made that takes into account factors such as detrimental effects on the environment, restoration of resources, and financial stability. Although existing decision-making strategies continuously failed to properly compensate for the basic challenges and inconsistencies arising from evaluating multiple factors problems, The existing techniques are impacted by a lack of ability to manage the complicated realities and disparities in specialist evaluations, the lack of clarity in facts, and the links between multiple decision factors. The activating imperative inspires this research effort to deliver an enhanced, secure, and flexible decision structure that can effectively navigate inconsistencies in FWM. Ordinary fuzzy decision-making techniques, despite being beneficial, frequently fail to demonstrate their ability to meet the challenges arising from pragmatically FWM cases, notably when given inconsistent opinions of experts and inaccurate or imprecise information.

This research is designed to encourage the implementation of prospective plans for inclusive growth by offering helpful information for beneficial and effective FWM behaviors. This study handles problems in this field by demonstrating a resistant and adaptable strategic structure conceived to alleviate apprehension and evaluate and determine FWTT. The q-ROFSS structure used for this research describes an intensely potent and proficient decision-making strategy that exceeds ordinary fuzzy set-based algorithms, which comprises FSS, IFSS, PyFS, PyFSS, and q-ROFS. This structure implements novel algorithms to alleviate the influence of unreliable or insecure information and demonstrate combinations during several selection criteria. This research executed a thorough evaluation of FWTT and listed significant shortcomings in the prevailing MAGDM strategy: no one extended Aczel–Alsina AOs in the q-ROFSS mechanism to evaluate the most efficient techniques for FWTT in the field of FWM. This study defines a novel decision-making mechanism built on the q-ROFSS structure, focused on efficiently handling MAGDM issues. The proposed technique presents an accurate and structured estimation of FWTT utilizing adjusted capabilities, which ensures intelligent and precise choices.

The conceptual justification for the significant value of this investigation is clarified as follows, presenting its intended impacts on both theoretical growth and authentic execution:

1. The q-ROFSS structure constitutes a malleable and precise product that describes convoluted and unreliable data. Its obvious directions are an objective foundation for regulating challenging information that promotes intelligent decision-making procedures. So, further research and modification of DM strategies in the q-ROFSS context is significant and pertinent.
2. Aczel–Alsina operations indicate valuable range through their exclusive parameterization, ensuring versatile transformations throughout decision-making. This study assesses the efficient realization of an innovative Aczel–Alsina operational law designed for the q-ROFSS context. The suggested approach increases decision consistency and precision using the necessary features of each operation.
3. MAGDM methods are renowned for their knowledge and performance in delivering solid and pragmatic outcomes. When Integrated with Aczel–Alsina–based operations, MAGDM approaches transmit extended tenacity and integrity relative to traditional methods, ensuring reliable decision-making procedures.
4. Even though the potential use of multiple MAGDM strategies, like TOPSIS, VIKOR, and EDAS, in distinctive fuzzy scenarios to assess and establish the most effective FWTT in FWM, significant deficiencies endure in the scientific literature about the execution of MAGDM structures exploiting q-ROFSS for FWTT selection. Determining a highly reliable treatment approach is a substantial obstacle, especially in emerging economies. The detailed nature of the selection procedure inside conventional MAGDM mechanisms mandates the formulation of an extended and credible technique. This research relieves these barriers by demonstrating an organized approach using q-ROFSS, which consistently seeks to observe the finest efficient FWTT.

The recommended approach is designed to resolve multiple crucial problems in the FWM industry. Also included are the following: In an FWM organization, what is the best and most beneficial way to reach decisions regarding determining an appropriate food waste treatment system? How can expertise gained from the abilities and opinions of FWM specialists be competently merged and carried out in the decision-making phase? How extensively will the Aczel–Alsina-based MAGDM strategy extend to develop an effective and organized system to choose the most favorable FWTT?

In the subsequent subsection, this study describes significant developments in managing obstacles.

### Contributions and layout of the study

1. This article expresses the execution and compliance of Aczel–Alsina AOs within the q-ROFSS structure. It describes the developments of two innovative operators: the q-ROFSAAWA and the q-ROFSAAWG operators. This research objectively evaluates and demonstrates multiple crucial aspects of these operators, such as idempotency, boundedness, and monotonicity, to maintain their reliability and impact in MAGDM structure.

2. An exceptional MAGDM algorithm is implemented, integrating a recently put forward Aczel–Alsina AOs in the q-ROFSS structure to select an optimum FW for a food company. The theoretical structure entails two significant phases, which include:

- The first stage is to collect and organize all necessary data using q-ROFSS-based Aczel–Alsina AOs to ensure precise and complete data representations.
- To facilitate the ability to analyze and identify the most beneficial decision, the next stage initiates a MAGDM method in the q-ROFSS context. This technique extends upon previous decision-making models by integrating the outlined method’s stability and adaptability, providing a unified strategy to manage convoluted decision-making challenges.

This technique investigates and determines the optimum FWTT approach in a wider FWM structure. This study competently meets the gaps in decision-making processes about food waste treatment and delivers an efficient, helpful resolution. The recommended method covers substantial comparison and sensitive analyses executed correctly and carefully. Our findings examine the accuracy and effectiveness of the strategy, demonstrating its expected implications for solving significant hurdles caused by determining and carrying out FWTT.

The overall structure of this research is described as follows: Section "Introduction and literature review" conveys the study’s strategic intentions, insisting on implementing divergences and imperfections in content frequently discovered in decision-making practices. It delivers a detailed review of existing literature on FWTT in distinct fuzzy structures. Section "Preliminaries" extensively investigates the important notions and conceptual frameworks that back this study. Section "Aczel–Alsina weighted average aggregation operators for q-rung orthopair fuzzy soft sets" demonstrates the Aczel–Alsina operational laws in the q-ROFSS structure, consequently delivering the q-ROFSAAWA and q-ROFSAAWG operators with a detailed analysis of their valuable aspects. Section "MAGDM model in q-rung orthopair fuzzy soft structure using Aczel–Alsina operators" describes the novel MAGDM technique using the proposed AOs. Section "Selection of the food waste treatment technology using the proposed MAGDM model" clarifies the real-world utilization of the described approach, exploiting analytical results to explain its adoption in FWM. Section “Sensitivity and comparative analysis” operates an exhaustive review to analyze the impact and practical implications of the constructed method. Verifying the credibility and receptivity of the stated strategy is the central objective of this section’s critical analysis of the tactics used. The methodology’s beneficial outcomes and potential advantages to FWM are described in the final section.

## Preliminaries

We will review some significant ideas more thoroughly in the subsequent section. That includes fuzzy sets, intuitionistic fuzzy sets, Pythagorean fuzzy sets, soft sets, Pythagorean fuzzy soft sets, and q-Rung orthopair fuzzy soft sets. This strong exposition creates strong logical research for the subsections that proceed.

### Definition 1

^31^ Let $L$ be a mapping such as $L: \left[\text{0,1}\right]\times \left[\text{0,1}\right]\to \left[\text{0,1}\right]$, then $L$ is said to be t-norm if the following properties are held:

1. $$L\left(u,v\right)=L\left(v,u\right)$$
2. $$L\left(u,v\right)=L\left(h,u\right) if v\le h$$
3. $$L\left(u,L\left(v,h\right)\right)=L\left(L\left(u,v\right),h\right)$$
4. $$L\left(u,1\right)=u$$

$$\forall ,u,v,h\in \left[\text{0,1}\right]$$.

### Definition 2

^31^ Let $M$ be a mapping such as $M: \left[\text{0,1}\right]\times \left[\text{0,1}\right]\to \left[\text{0,1}\right]$, then $M$ is said to be t-conorm if the following properties are held:

1. $$M\left(u,v\right)=M\left(v,u\right)$$
2. $$M\left(u,v\right)\le M\left(h,u\right)$$
3. $$M\left(u,M\left(v,h\right)\right)=M\left(M\left(u,v\right),h\right)$$
4. $$M\left(u,0\right)=u$$

$$\forall ,u,v,h\in \left[\text{0,1}\right]$$.

### Definition 3

^32^ Aczel–alsina t-norm and t-conorm can be defined as follows:

$$\left({L}_{A}^{\sigma }\right)\left(u,v\right)=\left\{\begin{array}{c}{L}_{D}\left(u,v\right) if \sigma =0\\ min\left(u,v\right) if \sigma =\infty \\ {e}^{-}{\left({\left(-ln\left(u\right)\right)}^{\sigma }+{\left(-ln\left(v\right)\right)}^{\sigma }\right)}^{\frac{1}{\sigma }} otherwise\end{array}\right\}$$

$$\left({M}_{A}^{\sigma }\right)\left(u,v\right)=\left\{\begin{array}{c}{M}_{D}\left(u,v\right) if \sigma =0\\ min\left(u,v\right) if \sigma =\infty \\ 1-{e}^{-}{\left({\left(-ln\left(u\right)\right)}^{\sigma }+{\left(-ln\left(v\right)\right)}^{\sigma }\right)}^{\frac{1}{\sigma }} otherwise\end{array}\right\}$$

where $$0\le \sigma \le \infty$$.

### Definition 4

^22^ A fuzzy set $A$ in a universe of discourse $W$ is defined as:

$$A = \left\{ {\left( {w_{i} ,u_{{A_{j} }} \left( {w_{i} } \right)} \right) \left| {w_{i} \in W} \right.} \right\}$$

where ${u}_{{A}_{j}}\left({w}_{i}\right)$ be the MD, such as ${u}_{{A}_{j}}\left({w}_{i}\right)\in [0, 1]$.

### Definition 5

^23^ An intuitionistic fuzzy set $A$ in a universe of discourse $W$ is defined as:

$$A = \left\{ {\left( {w_{i} ,\left( {u_{{A_{j} }} \left( {w_{i} } \right),v_{{A_{j} }} \left( {w_{i} } \right)} \right)} \right) \left| {w_{i} \in W} \right.} \right\}$$

where ${u}_{{A}_{j}}\left({w}_{i}\right)$ and ${v}_{{A}_{j}}\left({w}_{i}\right)$ be the MD and NMD, respectively, such as ${u}_{{A}_{j}}\left({w}_{i}\right),{v}_{{A}_{j}}\left({w}_{i}\right)\in \left[0, 1\right]$ and $0\le \left({u}_{{A}_{j}}\left({w}_{i}\right)\right)+\left({v}_{{A}_{j}}\left({w}_{i}\right)\right)\le 1$.

Integrating the resulting ideas into the previously described structure substantially enhanced its general structure and integrity. The structure’s functionality has been effective, permitting an extended examination of the problem’s related aspects.

### Definition 6

^24^ A Pythagorean fuzzy set $A$ in a universe of discourse $W$ is defined as:

$$A = \left\{ {\left( {w_{i} ,\left( {u_{{A_{j} }} \left( {w_{i} } \right),v_{{A_{j} }} \left( {w_{i} } \right)} \right)} \right) \left| {w_{i} \in W} \right.} \right\}$$

where ${u}_{{A}_{j}}\left({w}_{i}\right)$ and ${v}_{{A}_{j}}\left({w}_{i}\right)$ be the MD and NMD, respectively, such as ${u}_{{A}_{j}}\left({w}_{i}\right),{v}_{{A}_{j}}\left({w}_{i}\right)\in \left[0, 1\right]$ and $0\le {\left({u}_{{A}_{j}}\left({w}_{i}\right)\right)}^{2}+{\left({v}_{{A}_{j}}\left({w}_{i}\right)\right)}^{2}\le 1$.

### Definition 7

^25^ A q-rung orthopair fuzzy set $A$ in a universe of discourse $W$ is defined as:

$$A = \left\{ {\left( {w_{i} ,\left( {u_{{A_{j} }} \left( {w_{i} } \right),v_{{A_{j} }} \left( {w_{i} } \right)} \right)} \right) \left| {w_{i} \in W} \right.} \right\}$$

where ${u}_{{A}_{j}}\left({w}_{i}\right)$ and ${v}_{{A}_{j}}\left({w}_{i}\right)$ be the MD and NMD, respectively, such as ${u}_{{A}_{j}}\left({w}_{i}\right),{v}_{{A}_{j}}\left({w}_{i}\right)\in \left[0, 1\right]$ and $0\le {\left({u}_{{A}_{j}}\left({w}_{i}\right)\right)}^{q}+{\left({v}_{{A}_{j}}\left({w}_{i}\right)\right)}^{q}\le 1$, where $q\ge 3$.

This investigation focuses on a better approach to dealing with ambiguous and unclear data. Managing missing information and correcting data gaps can be approached from a new angle with the proposed methodology. Its main purpose is to improve data interpretation accuracy, dependability, and consistency in uncertain and complicated decision-making situations.

### Definition 8

^26^ Let $W$ and $\mathfrak{I}$ be a universe of discourse and attributes, $P(W)$ be the power set over $W$ and $A\subseteq \mathfrak{I}$. Then, a pair $\left(\beth , A\right)$ is called a soft set over $W$, where $\beth$ is a mapping:

$$\beth :A\to P(W)$$

Also, it can be defined as follows:

$$\left(\beth , A\right)=\left\{\beth \left(e\right)\in P(W):e\in \mathfrak{I}, \beth \left(e\right)= \varnothing if e\notin A\right\}$$

### Definition 9

^27^ Let $W$ and $\mathfrak{I}$ be a universe of discourse and attributes, $P(W)$ be the power set over $W$ and $A\subseteq \mathfrak{I}$. Then, a pair $\left(\beth , A\right)$ is called an IFSS over $W$.

$$\left( {\beth ,A} \right) = \left\{ {\left( {w_{i} ,\left( {u_{{A_{j} }} \left( {w_{i} } \right),v_{{A_{j} }} \left( {w_{i} } \right)} \right)} \right) \left| {w_{i} \in W} \right.} \right\}$$

where $\beth :A\to P(W)$ be a mapping and ${u}_{{A}_{j}}\left({w}_{i}\right)$ and ${v}_{{A}_{j}}\left({w}_{i}\right)$ be the MD and NMD, such as ${u}_{{A}_{j}}\left({w}_{i}\right),{v}_{{A}_{j}}\left({w}_{i}\right)\in \left[0, 1\right]$, $0\le {u}_{{A}_{j}}\left({w}_{i}\right),{v}_{{A}_{j}}\left({w}_{i}\right)\le 1$, such as $0\le {u}_{{A}_{j}}\left({w}_{i}\right)+{v}_{{A}_{j}}\left({w}_{i}\right)\le 1$.

### Definition 10

^28^ Let $W$ and $\mathfrak{I}$ be a universe of discourse and attributes, $P(W)$ be the power set over $W$ and $A\subseteq \mathfrak{I}$. Then, a pair $\left(\beth , A\right)$ is called a PyFSS over $W$.

$$\left( {\beth ,A} \right) = \left\{ {\left( {w_{i} ,\left( {u_{{A_{j} }} \left( {w_{i} } \right),v_{{A_{j} }} \left( {w_{i} } \right)} \right)} \right) \left| {w_{i} \in W} \right.} \right\}$$

where $\beth :A\to P(W)$ be a mapping and ${u}_{{A}_{j}}\left({w}_{i}\right)$, ${v}_{{A}_{j}}\left({w}_{i}\right)$ be the MD and NMD, such as ${u}_{{A}_{j}}\left({w}_{i}\right),{v}_{{A}_{j}}\left({w}_{i}\right)\in \left[0, 1\right]$, $0\le {u}_{{A}_{j}}\left({w}_{i}\right),{v}_{{A}_{j}}\left({w}_{i}\right)\le 1$ such as $0\le {\left({u}_{{A}_{j}}\left({w}_{i}\right)\right)}^{2}+{\left({v}_{{A}_{j}}\left({w}_{i}\right)\right)}^{2}\le 1$. Moreover, ${\pi }_{{A}_{j}}\left({w}_{i}\right)$ be the hesitancy of PyFSS, which can be defined as:

$${\pi }_{{A}_{j}}\left({w}_{i}\right)=\sqrt{1-{\left({u}_{{A}_{j}}\left({w}_{i}\right)\right)}^{2}-{\left({v}_{{A}_{j}}\left({w}_{i}\right)\right)}^{2}}$$

The $\beth_{{A_{j} }} \left( {w_{i} } \right) = \left\{ {\left( {w_{i} ,\left( {u_{{A_{j} }} \left( {w_{i} } \right),v_{{A_{j} }} \left( {w_{i} } \right)} \right)} \right) \left| {w_{i} \in W} \right.} \right\}$ can be written as $\beth_{A} = \left( {u_{A} ,v_{A} } \right)$ for readers convenience.

When ${u}_{{A}_{j}}\left({w}_{i}\right)+{v}_{{A}_{j}}\left({w}_{i}\right)>1$, traditional structures such as PyFSS^28^ are scarce for correctly labeling this form of facts. In such circumstances, the q-ROFSS framework, which amalgamates the distinct properties of q-ROFS and SS, delivers a proficient and accurate resolution. The q-ROFSS structure performs as an exceptional variant of q-ROFS, with improved capabilities for conquering various issues. It enables inspection of interruptions and fluctuations, allowing for more exact and authentic evaluations inside intricate databases. The q-ROFSS theory has proven its practical use in decision-making and statistical investigation, affirming its significance and efficacy in tackling complex and unclear issues.

### Definition 11

^29^ Let $W$ and $\mathfrak{I}$ be a universe of discourse and attributes, $P(W)$ be the power set over $W$ and $A\subseteq \mathfrak{I}$. Then, a pair $\left(\beth , A\right)$ is called a q-ROFSS over $W$.

$$\left( {\beth ,A} \right) = \left\{ {\left( {w_{i} ,\left( {u_{{A_{j} }} \left( {w_{i} } \right),v_{{A_{j} }} \left( {w_{i} } \right)} \right)} \right) \left| {w_{i} \in W} \right.} \right\}$$

where $\beth :A\to P(W)$ be a mapping and ${u}_{{A}_{j}}\left({w}_{i}\right)$, ${v}_{{A}_{j}}\left({w}_{i}\right)$ be the MD and NMD, such as ${u}_{{A}_{j}}\left({w}_{i}\right),{v}_{{A}_{j}}\left({w}_{i}\right)\in \left[0, 1\right]$, $0\le {u}_{{A}_{j}}\left({w}_{i}\right),{v}_{{A}_{j}}\left({w}_{i}\right)\le 1$ such as $0\le {\left({u}_{{A}_{j}}\left({w}_{i}\right)\right)}^{q}+{\left({v}_{{A}_{j}}\left({w}_{i}\right)\right)}^{q}\le 1$, where $q\ge 3$. Moreover, ${\pi }_{{A}_{j}}\left({w}_{i}\right)$ be the hesitancy of q-ROFSS, which can be defined as:

$${\pi }_{{A}_{j}}\left({w}_{i}\right)=\sqrt[q]{1-{\left({u}_{{A}_{j}}\left({w}_{i}\right)\right)}^{q}-{\left({v}_{{A}_{j}}\left({w}_{i}\right)\right)}^{q}}$$

### Definition 12

^29^ The score function promotes contrasting the results of two q-ROFSNs, assisting in determining the beneficial alternative from the collected information. Let ${\beth }_{A}=\left({u}_{A},{v}_{A}\right)$ denote a q-ROFSN; the score function for ${\beth }_{A}$ is formulated the following way:

1 $$S\left({\beth }_{A}\right)={\left({u}_{A}\right)}^{q}-{\left({v}_{A}\right)}^{q}+\left(\frac{{e}^{{\left({u}_{A}\right)}^{q}-{\left({v}_{A}\right)}^{q}}}{{e}^{{\left({u}_{A}\right)}^{q}-{\left({v}_{A}\right)}^{q}}+1}-\frac{1}{2}\right){\pi }^{q}$$

where $\pi =\sqrt[q]{1-\left({\left({u}_{A}\right)}^{q}+{\left({v}_{A}\right)}^{q}\right)}$ be the hesitancy of q-ROFSN and $S({\beth }_{A})\in [-\text{1,1}$]. Let ${\beth }_{{A}_{11}}=\left({u}_{{A}_{11}}, {v}_{{A}_{11}}\right)$ and ${\beth }_{{A}_{12}}=\left({u}_{{A}_{12}}, {v}_{{A}_{12}}\right)$ be two q-ROFSNs, then the comparison laws for q-ROFSNs are defined as follows:

If $\Psi \left({\beth }_{{A}_{11}}\right)>\Psi \left({\beth }_{{A}_{12}}\right)$, then we state ${\beth }_{{A}_{11}}\succcurlyeq {\beth }_{{A}_{12}}$.

If $\Psi \left({\beth }_{{A}_{11}}\right)<\Psi \left({\beth }_{{A}_{12}}\right)$, then we state ${\beth }_{{A}_{11}}\preccurlyeq {\beth }_{{A}_{12}}$.

If $$\Psi \left({\beth }_{{A}_{11}}\right)=\Psi \left({\beth }_{{A}_{12}}\right)$$, then $${\beth }_{{A}_{11}}={\beth }_{{A}_{12}}$$

If ${\pi }_{{A}_{11}}>{\pi }_{{A}_{12}}$, then we state ${\beth }_{{A}_{11}}<{\beth }_{{A}_{12}}$

If ${\pi }_{{A}_{11}}<{\pi }_{{A}_{12}}$, then we state ${\beth }_{{A}_{11}}>{\beth }_{{A}_{12}}$.

The efficient formation of information is necessary to ensure its integrity and validity in decision-making. This subsection provides a novel strategy for implementing facts into the q-ROFSS structure, eliminating the challenges of preceding studies that exclusively focus on AOs for q-ROFS-based facts. This investigation incorporates AA-TNs and AA-TCNs to overcome accumulation obstacles competently and eventually enhance the standard and stability of opinions based on q-ROFSS data.

## Aczel–Alsina weighted average aggregation operators for q-rung orthopair fuzzy soft sets

This paper consists of basic steps for handling and performing data analysis expressed as q-ROFSVs, contingent on the AA-TNMs and AA-TCNMs. These operational rules produce a systematic structure for an organization’s collection and application of considerable q-ROFS data to support managerial and decision-making environments. We evaluate and utilize Aczel–Alsina AOs presented for q-ROFSVs to emphasize these fundamental concepts. This investigation develops two principal operators: the q-ROFSAAWA and the q-ROFSAAWG operators. These operators were developed to boost the aggregation procedure’s efficacy and accuracy, facilitating enhanced data integration. Also, we determine and ensure the core features of these operators, such as idempotency, boundedness, and monotonicity. A rigorous review of these aspects justifies the resilience and cognitive integrity of the stated operators, maintaining their reliability over diverse decision-making circumstances.

### Definition 13

Let ${\beth }_{A}=\left({u}_{A}, {v}_{A}\right)$, ${\beth }_{{A}_{11}}=\left({u}_{{A}_{11}}, {v}_{{A}_{11}}\right)$ and ${\beth }_{{A}_{12}}=\left({u}_{{A}_{12}}, {v}_{{A}_{12}}\right)$, be the q-ROFSVs. Then, the Aczel–Alsina algebraic operational laws are defined as follows:

1. $${\beth }_{{A_{{11}} }} \oplus {\beth }_{{A_{{12}} }} = \left( {\begin{array}{*{20}c} {\sqrt[q]{{1 - e^{{ - \left( {\left( { - \ln \left( {1 - \left( {u_{{A_{{11}} }} } \right)^{q} } \right)} \right)^{\tau } + \left( { - \ln \left( {1 - \left( {u_{{A_{{12}} }} } \right)^{q} } \right)^{\tau } } \right)} \right)^{{\frac{1}{\tau }}} }} }},e^{{ - \left( {\left( { - \ln \left( {v_{{A_{{11}} }} } \right)} \right)^{\tau } + \left( { - \ln \left( {v_{{A_{{12}} }} } \right)} \right)^{\tau } } \right)^{{^{{\frac{1}{\tau }}} }} }} } \\ \end{array} } \right)$$

2. $${\beth }_{{A_{{11}} }} \otimes {\beth }_{{A_{{12}} }} = \left( {\begin{array}{*{20}c} {e^{{ - \left( {\left( { - \ln \left( {u_{{A_{{11}} }} } \right)} \right)^{\tau } + \left( { - \ln \left( {u_{{A_{{12}} }} } \right)} \right)^{\tau } } \right)^{{\frac{1}{\tau }}} }} ,\sqrt[q]{{1 - e^{{ - \left( {\left( { - \ln \left( {1 - \left( {v_{{A_{{11}} }} } \right)^{q} } \right)} \right)} \right)^{\tau } + \left( { - \ln \left( {1 - \left( {v_{{A_{{12}} }} } \right)^{q} } \right)^{\tau } } \right)^{{\frac{1}{\tau }}} }} }}} \\ \end{array} } \right)$$

3. $$\delta .\beth_{A} = \left( {\begin{array}{*{20}c} {\sqrt[q]{{1 - e^{{ - \left( {\delta *\left( { - ln\left( {1 - \left( {u_{A} } \right)^{q} } \right)} \right)^{\tau } } \right)^{{\frac{1}{\tau }}} }} }},e^{{ - \left( {\delta \left( { - ln\left( {v_{A} } \right)} \right)^{\tau } } \right)^{{\frac{1}{\tau }}} }} } \\ \end{array} } \right)$$

4. $$\beth_{A}^{\delta } = \left( {\begin{array}{*{20}c} {e^{{ - \left( {\delta \left( { - ln\left( {u_{A} } \right)} \right)^{\tau } } \right)^{{\frac{1}{\tau }}} }} , \sqrt[q]{{1 - e^{{ - \left( {\delta \left( { - ln\left( {1 - \left( {v_{A} } \right)^{q} } \right)} \right)^{\tau } } \right)^{{\frac{1}{\tau }}} }} }}} \\ \end{array} } \right)$$

where $$q\ge 3$$, $$\tau \ge 1$$ and $$\delta >0$$.

### Theorem 1

*Let* ${\beth }_{A}=\left({u}_{A}, {v}_{A}\right)$, ${\beth }_{{A}_{11}}=\left({u}_{{A}_{11}}, {v}_{{A}_{11}}\right)$ *and* ${\beth }_{{A}_{12}}=\left({u}_{{A}_{12}}, {v}_{{A}_{12}}\right)$, *be the q-ROFSVs.* *Then, the following properties are held*:

1. $$\beth_{{A_{11} }} \oplus \beth_{{A_{12} }} = \beth_{{A_{12} }} \oplus \beth_{{A_{11} }}$$
2. $$\beth_{{A_{11} }} \otimes \beth_{{A_{12} }} = \beth_{{A_{12} }} \otimes \beth_{{A_{11} }}$$
3. $$\delta \left( {\beth_{{A_{11} }} \oplus \beth_{{A_{12} }} } \right) = \delta \beth_{{A_{11} }} \delta \beth_{{A_{12} }}$$
4. $$\left( {\delta_{1} \oplus \delta_{2} } \right)\beth_{{A_{ij} }} = \delta_{1} \beth_{{A_{ij} }} \oplus \delta_{2} \beth_{{A_{ij} }}$$
5. $$\left( {\beth_{{A_{11} }} \otimes \beth_{{A_{12} }} } \right)^{\delta } = \beth_{{A_{11} }}^{\delta } \otimes \beth_{{A_{11} }}^{\delta }$$
6. $$\beth_{{A_{ij} }}^{{\delta_{1} }} \otimes \beth_{{A_{ij} }}^{{\delta_{2} }} = \beth_{{A_{ij} }}^{{\left( {\delta_{1} + \delta_{2} } \right)}}$$

*where* $$\delta , {\delta }_{1},{\delta }_{2}>0$$.

### Proof

The proof is straightforward.

### Definition 14

Let ${\beth }_{{A}_{ij}}=\left({u}_{{A}_{ij}}, {v}_{{A}_{ij}}\right)$ be a collection of q-ROFSVs, where $i,j=\text{1,2},\dots \dots .n,m$. Then, a q-ROFSAAWA is defined as:

$$\begin{aligned} & q - ROFSAAWA\left( {\beth_{{A_{11} }} ,\beth_{{A_{12} }} , \ldots \ldots \beth_{{A_{nm} }} } \right) \\ & = o_{1} {\mathfrak{H}}_{1} \beth_{{A_{11} }} \oplus o_{1} {\mathfrak{H}}_{2} \beth_{{A_{12} }} \oplus \ldots \ldots \oplus o_{m} {\mathfrak{H}}_{n} \beth_{{A_{nm} }} = \left( {\mathop {\mathop \oplus \limits_{j = 1} }\limits^{m} o_{j} \left( {\mathop {\mathop \oplus \limits_{i = 1} }\limits^{n} {\mathfrak{H}}_{i} \beth_{{A_{ij} }} } \right)} \right) \\ \end{aligned}$$

where ${\mathfrak{H}}_{i}$ and ${\calligra{\rotatebox[origin=c]{22}{o}}}_{j}$ be the weights of experts and attributes such as ${\mathfrak{H}}_{i}>0,\sum_{i=1}^{n}{\mathfrak{H}}_{i}=1$ and ${\calligra{\rotatebox[origin=c]{22}{o}}}_{j}>0,\sum_{j=1}^{m}{\calligra{\rotatebox[origin=c]{22}{o}}}_{j}=1$.

### Theorem 2

*Let* ${\beth }_{{A}_{ij}}=\left({u}_{{A}_{ij}}, {v}_{{A}_{ij}}\right)$ *be a collection of* *q-ROFSVs* *where* $i,j=\text{1,2},\dots \dots .n,m$. *The compiled outcome extracted by the* *q-ROFSAAWA operator consequently appears as a q-ROFSV*.

2 $$\begin{aligned} & q - ROFSAAWA\left( {{\beth }_{{A_{{11}} }} ,{\beth }_{{A_{{12}} }} , \ldots \ldots {\beth }_{{A_{{nm}} }} } \right) \\ & \quad = \left( {\begin{array}{*{20}c} {\sqrt[q]{{1 - e^{{ - \left( {\sum\limits_{{j = 1}}^{m} {\calligra{\rotatebox[origin=c]{22}{o}}}_{j} \left( { - ln\left( {e^{{ - \left( {\left( {\sum\limits_{{i = 1}}^{n} {{\mathfrak{H}}_{i} } \left( { - ln\left( {1 - \left( {u_{{A_{{ij}} }} } \right)^{q} } \right)} \right)^{\tau } } \right)^{{\frac{1}{\tau }}} } \right)}} } \right)} \right)^{\tau } } \right)^{{\frac{1}{\tau }}} }} }},e^{{ - \left( {\sum\limits_{{j = 1}}^{m} {\calligra{\rotatebox[origin=c]{22}{o}}}_{j} \left( { - ln\left( {e^{{ - \left( {\sum\limits_{{i = 1}}^{n} {{\mathfrak{H}}_{i} } \left( { - ln\left( {v_{{A_{{ij}} }} } \right)} \right)^{\tau } } \right)^{{\frac{1}{\tau }}} }} } \right)} \right)^{\tau } } \right)^{{\frac{1}{\tau }}} }} } \\ \end{array} } \right) \\ \end{aligned}$$

*where* ${\mathfrak{H}}_{i}$ *and* ${\calligra{\rotatebox[origin=c]{22}{o}}}_{j}$ *be the weights of experts and attributes such as* ${\mathfrak{H}}_{i}>0,\sum_{i=1}^{n}{\mathfrak{H}}_{i}=1$ *and* ${\calligra{\rotatebox[origin=c]{22}{o}}}_{j}>0,\sum_{j=1}^{m}{\calligra{\rotatebox[origin=c]{22}{o}}}_{j}=1$.

### Proof

Using the mathematical induction method, we can show that Theorem 2 is true in the following way:

If $n=2$ and $m=2$, then by using our defined aczel–alsina operations on IVq-ROFSS we obtain:

$${\calligra{\rotatebox[origin=c]{22}{o}}}_{1}{\mathfrak{H}}_{1}{{\beth }}_{{A}_{11}}=\left(\begin{array}{c}\sqrt[q]{1-{e}^{-{\left({\calligra{\rotatebox[origin=c]{22}{o}}}_{1}{\left(-ln\left({e}^{-\left({\left({\mathfrak{H}}_{1}{\left(-ln\left(1-{\left({u}_{{A}_{11}}\right)}^{q}\right)\right)}^{\tau }\right)}^{\frac{1}{\tau }}\right)}\right)\right)}^{\tau }\right)}^{\frac{1}{\tau }}}},{e}^{-{\left({{\calligra{\rotatebox[origin=c]{22}{o}}}_{1}\left(-ln\left({e}^{-{\left({\mathfrak{H}}_{1}{\left(-ln\left({v}_{{A}_{11}}\right)\right)}^{\tau }\right)}^{\frac{1}{\tau }}}\right)\right)}^{\tau }\right)}^{\frac{1}{\tau }}}\end{array}\right),$$

$${\calligra{\rotatebox[origin=c]{22}{o}}}_{2}{\mathfrak{H}}_{1}{{\beth }}_{{A}_{12}}=\left(\begin{array}{c}\sqrt[q]{1-{e}^{-{\left({\calligra{\rotatebox[origin=c]{22}{o}}}_{2}{\left(-ln\left({e}^{-\left({\left({\mathfrak{H}}_{1}{\left(-ln\left(1-{\left({u}_{{A}_{12}}\right)}^{q}\right)\right)}^{\tau }\right)}^{\frac{1}{\tau }}\right)}\right)\right)}^{\tau }\right)}^{\frac{1}{\tau }}}},{e}^{-{\left({{\calligra{\rotatebox[origin=c]{22}{o}}}_{2}\left(-ln\left({e}^{-{\left({\mathfrak{H}}_{1}{\left(-ln\left({v}_{{A}_{12}}\right)\right)}^{\tau }\right)}^{\frac{1}{\tau }}}\right)\right)}^{\tau }\right)}^{\frac{1}{\tau }}}\end{array}\right),$$

$${\calligra{\rotatebox[origin=c]{22}{o}}}_{1}{\mathfrak{H}}_{2}{{\beth }}_{{A}_{21}}=\left(\begin{array}{c}\sqrt[q]{1-{e}^{-{\left({\calligra{\rotatebox[origin=c]{22}{o}}}_{1}{\left(-ln\left({e}^{-\left({\left({\mathfrak{H}}_{2}{\left(-ln\left(1-{\left({u}_{{A}_{21}}\right)}^{q}\right)\right)}^{\tau }\right)}^{\frac{1}{\tau }}\right)}\right)\right)}^{\tau }\right)}^{\frac{1}{\tau }}}},{e}^{-{\left({{\calligra{\rotatebox[origin=c]{22}{o}}}_{1}\left(-ln\left({e}^{-{\left({\mathfrak{H}}_{2}{\left(-ln\left({v}_{{A}_{21}}\right)\right)}^{\tau }\right)}^{\frac{1}{\tau }}}\right)\right)}^{\tau }\right)}^{\frac{1}{\tau }}}\end{array}\right),$$

$${\calligra{\rotatebox[origin=c]{22}{o}}}_{2}{\mathfrak{H}}_{2}{{\beth }}_{{A}_{22}}=\left(\begin{array}{c}\sqrt[q]{1-{e}^{-{\left({\calligra{\rotatebox[origin=c]{22}{o}}}_{2}{\left(-ln\left({e}^{-\left({\left({\mathfrak{H}}_{2}{\left(-ln\left(1-{\left({u}_{{A}_{22}}\right)}^{q}\right)\right)}^{\tau }\right)}^{\frac{1}{\tau }}\right)}\right)\right)}^{\tau }\right)}^{\frac{1}{\tau }}}},{e}^{-{\left({{\calligra{\rotatebox[origin=c]{22}{o}}}_{2}\left(-ln\left({e}^{-{\left({\mathfrak{H}}_{2}{\left(-ln\left({v}_{{A}_{22}}\right)\right)}^{\tau }\right)}^{\frac{1}{\tau }}}\right)\right)}^{\tau }\right)}^{\frac{1}{\tau }}}\end{array}\right).$$

Based on Definition 13, we obtain,

$$q-ROFSAAWA\left({\beth }_{{A}_{11}},{\beth }_{{A}_{12}},{\beth }_{{A}_{21}}, {\beth }_{{A}_{22}}\right)={\calligra{\rotatebox[origin=c]{22}{o}}}_{1}{\mathfrak{H}}_{1}{\beth }_{{A}_{11}}\oplus{\calligra{\rotatebox[origin=c]{22}{o}}}_{2}{\mathfrak{H}}_{1}{\beth }_{{A}_{12}}\oplus{\calligra{\rotatebox[origin=c]{22}{o}}}_{1}{\mathfrak{H}}_{2}{\beth }_{{A}_{21}}\oplus{\calligra{\rotatebox[origin=c]{22}{o}}}_{2}{\mathfrak{H}}_{2}{\beth }_{{A}_{22}}$$

$$=\left(\begin{array}{c}\sqrt[q]{1-{e}^{-{\left({\calligra{\rotatebox[origin=c]{22}{o}}}_{1}{\left(-ln\left({e}^{-\left({\left({\mathfrak{H}}_{1}{\left(-ln\left(1-{\left({u}_{{A}_{11}}\right)}^{q}\right)\right)}^{\tau }\right)}^{\frac{1}{\tau }}\right)}\right)\right)}^{\tau }\right)}^{\frac{1}{\tau }}}},\\ {e}^{-{\left({{\calligra{\rotatebox[origin=c]{22}{o}}}_{1}\left(-ln\left({e}^{-{\left({\mathfrak{H}}_{1}{\left(-ln\left({v}_{{A}_{11}}\right)\right)}^{\tau }\right)}^{\frac{1}{\tau }}}\right)\right)}^{\tau }\right)}^{\frac{1}{\tau }}}\end{array}\right)\oplus\left(\begin{array}{c}\sqrt[q]{1-{e}^{-{\left({\calligra{\rotatebox[origin=c]{22}{o}}}_{2}{\left(-ln\left({e}^{-\left({\left({\mathfrak{H}}_{1}{\left(-ln\left(1-{\left({u}_{{A}_{12}}\right)}^{q}\right)\right)}^{\tau }\right)}^{\frac{1}{\tau }}\right)}\right)\right)}^{\tau }\right)}^{\frac{1}{\tau }}}},\\ {e}^{-{\left({{\calligra{\rotatebox[origin=c]{22}{o}}}_{2}\left(-ln\left({e}^{-{\left({\mathfrak{H}}_{1}{\left(-ln\left({v}_{{A}_{12}}\right)\right)}^{\tau }\right)}^{\frac{1}{\tau }}}\right)\right)}^{\tau }\right)}^{\frac{1}{\tau }}}\end{array}\right)$$

$$\oplus\left(\begin{array}{c}\sqrt[q]{1-{e}^{-{\left({\calligra{\rotatebox[origin=c]{22}{o}}}_{1}{\left(-ln\left({e}^{-\left({\left({\mathfrak{H}}_{2}{\left(-ln\left(1-{\left({u}_{{A}_{21}}\right)}^{q}\right)\right)}^{\tau }\right)}^{\frac{1}{\tau }}\right)}\right)\right)}^{\tau }\right)}^{\frac{1}{\tau }}}},\\ {e}^{-{\left({{\calligra{\rotatebox[origin=c]{22}{o}}}_{1}\left(-ln\left({e}^{-{\left({\mathfrak{H}}_{2}{\left(-ln\left({v}_{{A}_{21}}\right)\right)}^{\tau }\right)}^{\frac{1}{\tau }}}\right)\right)}^{\tau }\right)}^{\frac{1}{\tau }}}\end{array}\right)\oplus\left(\begin{array}{c}\sqrt[q]{1-{e}^{-{\left({\calligra{\rotatebox[origin=c]{22}{o}}}_{2}{\left(-ln\left({e}^{-\left({\left({\mathfrak{H}}_{2}{\left(-ln\left(1-{\left({u}_{{A}_{22}}\right)}^{q}\right)\right)}^{\tau }\right)}^{\frac{1}{\tau }}\right)}\right)\right)}^{\tau }\right)}^{\frac{1}{\tau }}}},\\ {e}^{-{\left({{\calligra{\rotatebox[origin=c]{22}{o}}}_{2}\left(-ln\left({e}^{-{\left({\mathfrak{H}}_{2}{\left(-ln\left({v}_{{A}_{22}}\right)\right)}^{\tau }\right)}^{\frac{1}{\tau }}}\right)\right)}^{\tau }\right)}^{\frac{1}{\tau }}}\end{array}\right)$$

$$=\left(\begin{array}{c}\sqrt[q]{1-{e}^{-{\left(\sum_{j=1}^{2}{\calligra{\rotatebox[origin=c]{22}{o}}}_{j}{\left(-ln\left({e}^{-\left({\left(\sum_{i=1}^{2}{\mathfrak{H}}_{i}{\left(-ln\left(1-{\left({u}_{{A}_{ij}}\right)}^{q}\right)\right)}^{\tau }\right)}^{\frac{1}{\tau }}\right)}\right)\right)}^{\tau }\right)}^{\frac{1}{\tau }}}},{e}^{-{\left({\sum_{j=1}^{2}{\calligra{\rotatebox[origin=c]{22}{o}}}_{j}\left(-ln\left({e}^{-{\left(\sum_{i=1}^{2}{\mathfrak{H}}_{i}{\left(-ln\left({v}_{{A}_{ij}}\right)\right)}^{\tau }\right)}^{\frac{1}{\tau }}}\right)\right)}^{\tau }\right)}^{\frac{1}{\tau }}}\end{array}\right)$$

Hence, it is true for $n=2$ and $m=2$.

Suppose Eq. 2 is hold for $n=k$ and $m={\ell}$. Then,

$$\begin{aligned} & q - ROFSAAWA\left( {{\beth }_{{A_{{11}} }} ,{\beth }_{{A_{{12}} }} , \ldots \ldots {\beth }_{{A_{{k\ell }} }} } \right) \\ & \quad = \left( {\begin{array}{*{20}c} {\sqrt[q]{{1 - e^{{ - \left( {\sum\limits_{{j = 1}}^{\ell } {\calligra{\rotatebox[origin=c]{22}{o}}}_{j} \left( { - ln\left( {e^{{ - \left( {\left( {\sum\limits_{{i = 1}}^{k} {{\mathfrak{H}}_{i} } \left( { - ln\left( {1 - \left( {u_{{A_{{ij}} }} } \right)^{q} } \right)} \right)^{\tau } } \right)^{{\frac{1}{\tau }}} } \right)}} } \right)} \right)^{\tau } } \right)^{{\frac{1}{\tau }}} }} }},e^{{ - \left( {\sum\limits_{{j = 1}}^{\ell } {\calligra{\rotatebox[origin=c]{22}{o}}}_{j} \left( { - ln\left( {e^{{ - \left( {\sum\limits_{{i = 1}}^{k} {{\mathfrak{H}}_{i} } \left( { - ln\left( {v_{{A_{{ij}} }} } \right)} \right)^{\tau } } \right)^{{\frac{1}{\tau }}} }} } \right)} \right)^{\tau } } \right)^{{\frac{1}{\tau }}} }} } \\ \end{array} } \right) \\ \end{aligned}$$

For $$n=k+1$$ and $$m={\ell}+1$$.

$$q-ROFSAAWA\left({\beth }_{{A}_{11}},{\beth }_{{A}_{12}},\dots \dots {\beth }_{{A}_{\left(k+1\right)\left({\ell}+1\right)}}\right)=\stackrel{m}{\underset{j=1}{\oplus}}{\calligra{\rotatebox[origin=c]{22}{o}}}_{j}\left(\stackrel{n}{\underset{i=1}{\oplus}}{\mathfrak{H}}_{i}{\beth }_{{A}_{ij}}\right)\oplus{\calligra{\rotatebox[origin=c]{22}{o}}}_{({\ell}+1)}\left({\mathfrak{H}}_{(k+1)}{\beth }_{{A}_{(k+1)({\ell}+1)}}\right)$$

As we know that

$$\begin{aligned} & \mathop {\mathop \oplus \limits_{{j = 1}} }\limits^{m} {\calligra{\rotatebox[origin=c]{22}{o}}}_{j} \left( {\mathop {\mathop \oplus \limits_{{i = 1}} }\limits^{n} {\mathfrak{H}}_{i} {\beth }_{{A_{{ij}} }} } \right) \\ & \quad = \left( {\begin{array}{*{20}c} {\sqrt[q]{{1 - e^{{ - \left( {\sum\limits_{{j = 1}}^{m} {\calligra{\rotatebox[origin=c]{22}{o}}}_{j} \left( { - ln\left( {e^{{ - \left( {\left( {\sum\limits_{{i = 1}}^{n} {{\mathfrak{H}}_{i} } \left( { - ln\left( {1 - \left( {u_{{A_{{ij}} }} } \right)^{q} } \right)} \right)^{\tau } } \right)^{{\frac{1}{\tau }}} } \right)}} } \right)} \right)^{\tau } } \right)^{{\frac{1}{\tau }}} }} }},e^{{ - \left( {\sum\limits_{{j = 1}}^{m} {\calligra{\rotatebox[origin=c]{22}{o}}}_{j} \left( { - ln\left( {e^{{ - \left( {\sum\limits_{{i = 1}}^{n} {{\mathfrak{H}}_{i} } \left( { - ln\left( {v_{{A_{{ij}} }} } \right)} \right)^{\tau } } \right)^{{\frac{1}{\tau }}} }} } \right)} \right)^{\tau } } \right)^{{\frac{1}{\tau }}} }} } \\ \end{array} } \right) \\ \end{aligned}$$

and

$$\begin{aligned} & {\calligra{\rotatebox[origin=c]{22}{o}}}_{{(\ell + 1)}} \left( {{\mathfrak{H}}_{{(k + 1)}} {\beth }_{{A_{{(k + 1)(\ell + 1)}} }} } \right) \\ & \quad = \left( {\begin{array}{*{20}c} {\sqrt[q]{{1 - e^{{ - \left( {{\calligra{\rotatebox[origin=c]{22}{o}}}_{{(\ell + 1)}} \left( { - ln\left( {e^{{ - \left( {\left( {{\mathfrak{H}}_{{(k + 1)}} \left( { - ln\left( {1 - \left( {u_{{A_{{(k + 1)(\ell + 1)}} }} } \right)^{q} } \right)} \right)^{\tau } } \right)^{{\frac{1}{\tau }}} } \right)}} } \right)} \right)^{\tau } } \right)^{{\frac{1}{\tau }}} }} }},e^{{ - \left( {{\calligra{\rotatebox[origin=c]{22}{o}}}_{{(\ell + 1)}} \left( { - ln\left( {e^{{ - \left( {{\mathfrak{H}}_{{(k + 1)}} \left( { - ln\left( {v_{{A_{{(k + 1)(\ell + 1)}} }} } \right)} \right)^{\tau } } \right)^{{\frac{1}{\tau }}} }} } \right)} \right)^{\tau } } \right)^{{\frac{1}{\tau }}} }} } \\ \end{array} } \right) \\ \end{aligned}$$

Then,

$$\begin{aligned} & q - ROFSAAWA\left( {{\beth }_{{A_{{11}} }} ,{\beth }_{{A_{{12}} }} , \ldots \ldots {\beth }_{{A_{{\left( {k + 1} \right)\left( {\ell + 1} \right)}} }} } \right) \\ &\quad = \mathop {\mathop \oplus \limits_{{j = 1}} }\limits^{m} {\calligra{\rotatebox[origin=c]{22}{o}}}_{j} \left( {\mathop {\mathop \oplus \limits_{{i = 1}} }\limits^{n} {\mathfrak{H}}_{i} {\beth }_{{A_{{ij}} }} } \right) \oplus {\calligra{\rotatebox[origin=c]{22}{o}}}_{{\left( {\ell + 1} \right)}} \left( {{\mathfrak{H}}_{{\left( {k + 1} \right)}} {\beth }_{{A_{{\left( {k + 1} \right)\left( {\ell + 1} \right)}} }} } \right) \\ & \quad = \left( {\begin{array}{*{20}c} {\sqrt[q]{{1 - e^{{ - \left( {\sum\limits_{{j = 1}}^{\ell } {{\calligra{\rotatebox[origin=c]{22}{o}}}_{j} } \left( { - ln\left( {e^{{ - \left( {\left( {\sum\limits_{{i = 1}}^{k} {{\mathfrak{H}}_{i} } \left( { - ln\left( {1 - \left( {u_{{A_{{ij}} }} } \right)^{q} } \right)} \right)^{\tau } } \right)^{{\frac{1}{\tau }}} } \right)}} } \right)} \right)^{\tau } } \right)^{{\frac{1}{\tau }}} }} }},} \\ {e^{{ - \left( {\sum\limits_{{j = 1}}^{\ell } {{\calligra{\rotatebox[origin=c]{22}{o}}}_{j} } \left( { - ln\left( {e^{{ - \left( {\sum\limits_{{i = 1}}^{k} {{\mathfrak{H}}_{i} } \left( { - ln\left( {v_{{A_{{ij}} }} } \right)} \right)^{\tau } } \right)^{{\frac{1}{\tau }}} }} } \right)} \right)^{\tau } } \right)^{{\frac{1}{\tau }}} }} } \\ \end{array} } \right) \\& \quad \oplus \left( {\begin{array}{*{20}c} {\sqrt[q]{{1 - e^{{ - \left( {{\calligra{\rotatebox[origin=c]{22}{o}}}_{{\left( {\ell + 1} \right)}} \left( { - ln\left( {e^{{ - \left( {\left( {{\mathfrak{H}}_{{\left( {k + 1} \right)}} \left( { - ln\left( {1 - \left( {u_{{A_{{\left( {k + 1} \right)\left( {\ell + 1} \right)}} }} } \right)^{q} } \right)} \right)^{\tau } } \right)^{{\frac{1}{\tau }}} } \right)}} } \right)} \right)^{\tau } } \right)^{{\frac{1}{\tau }}} }} }},} \\ {e^{{ - \left( {{\calligra{\rotatebox[origin=c]{22}{o}}}_{{(\ell + 1)}} \left( { - ln\left( {e^{{ - \left( {{\mathfrak{H}}_{{(k + 1)}} \left( { - ln\left( {v_{{A_{{(k + 1)(\ell + 1)}} }} } \right)} \right)^{\tau } } \right)^{{\frac{1}{\tau }}} }} } \right)} \right)^{\tau } } \right)^{{\frac{1}{\tau }}} }} } \\ \end{array} } \right) \\ \end{aligned}$$

$$=\left(\begin{array}{c}\sqrt[q]{1-{e}^{-{\left(\sum_{j=1}^{{\ell}+1}{\calligra{\rotatebox[origin=c]{22}{o}}}_{j}{\left(-ln\left({e}^{-\left({\left(\sum_{i=1}^{k+1}{\mathfrak{H}}_{i}{\left(-ln\left(1-{\left({u}_{{A}_{ij}}\right)}^{q}\right)\right)}^{\tau }\right)}^{\frac{1}{\tau }}\right)}\right)\right)}^{\tau }\right)}^{\frac{1}{\tau }}}},{e}^{-{\left({\sum_{j=1}^{{\ell}+1}{\calligra{\rotatebox[origin=c]{22}{o}}}_{j}\left(-ln\left({e}^{-{\left(\sum_{i=1}^{k+1}{\mathfrak{H}}_{i}{\left(-ln\left({v}_{{A}_{ij}}\right)\right)}^{\tau }\right)}^{\frac{1}{\tau }}}\right)\right)}^{\tau }\right)}^{\frac{1}{\tau }}}\end{array}\right)$$

Which represents that Eq. 3.1 satisfied for *n* = *k* + 1 and *m* = ℓ + 1. Hence, it is proved that Eq. 3.1 holds $\forall$ $n,m>0$.

### Proposition 1

*If* ${\beth }_{{A}_{ij}}=\left({u}_{{A}_{ij}}, {v}_{{A}_{ij}}\right)$ *be a collection of* *q-ROFSVs where* $i,j=\text{1,2},\dots \dots .n,m$ *and* ${\mathfrak{H}}_{i}$, ${\calligra{\rotatebox[origin=c]{22}{o}}}_{j}$ *be the weights of experts and attributes, respectively, such as* ${\mathfrak{H}}_{i}>0,\sum_{i=1}^{n}{\mathfrak{H}}_{i}=1$ *and* ${\calligra{\rotatebox[origin=c]{22}{o}}}_{j}>0,\sum_{j=1}^{m}{\calligra{\rotatebox[origin=c]{22}{o}}}_{j}=1$.

### Idempotency

Let ${\beth }_{{A}_{ij}}={\beth }_{{A}_{o}}=\left({u}_{{A}_{o}}, {v}_{{A}_{o}}\right)$ is satisfied for any $i=1, 2, 3, \dots , n;j=1, 2, 3, \dots ,m$. Then,

$$q-ROFSAAWA\left({\beth }_{{A}_{11}},{\beth }_{{A}_{12}},\dots \dots {\beth }_{{A}_{nm}}\right)={\beth }_{{A}_{o}}$$

#### Proof

As we know that ${\beth }_{{A}_{ij}}=\left({u}_{{A}_{ij}}, {v}_{{A}_{ij}}\right)$ be a collection of q-ROFSVs and

$$\begin{aligned} & q - ROFSAAWA\left( {{\beth }_{{A_{{11}} }} ,{\beth }_{{A_{{12}} }} , \ldots \ldots {\beth }_{{A_{{nm}} }} } \right) \\ & \quad = \left( {\begin{array}{*{20}c} {\sqrt[q]{{1 - e^{{ - \left( {\sum\limits_{{j = 1}}^{m} {{\calligra{\rotatebox[origin=c]{22}{o}}}_{j} } \left( { - ln\left( {e^{{ - \left( {\left( {\sum\limits_{{i = 1}}^{n} {{\mathfrak{H}}_{i} } \left( { - ln\left( {1 - \left( {u_{{A_{{ij}} }} } \right)^{q} } \right)} \right)^{\tau } } \right)^{{\frac{1}{\tau }}} } \right)}} } \right)} \right)^{\tau } } \right)^{{\frac{1}{\tau }}} }} }},e^{{ - \left( {\sum\limits_{{j = 1}}^{m} {{\calligra{\rotatebox[origin=c]{22}{o}}}_{j} } \left( { - ln\left( {e^{{ - \left( {\sum\limits_{{i = 1}}^{n} {{\mathfrak{H}}_{i} } \left( { - ln\left( {v_{{A_{{ij}} }} } \right)} \right)^{\tau } } \right)^{{\frac{1}{\tau }}} }} } \right)} \right)^{\tau } } \right)^{{\frac{1}{\tau }}} }} } \\ \end{array} } \right) \\ \end{aligned}$$

Since ${\beth }_{{A}_{o}}=\left({u}_{{A}_{o}}, {v}_{{A}_{o}}\right)$, so the above equation is reduced to

$$\begin{aligned} & q - ROFSAAWA\left( {{\beth }_{{A_{{11}} }} ,{\beth }_{{A_{{12}} }} , \ldots \ldots {\beth }_{{A_{{nm}} }} } \right) \\ & \quad = \left( {\begin{array}{*{20}c} {\sqrt[q]{{1 - e^{{ - \left( {\sum\limits_{{j = 1}}^{m} {{\calligra{\rotatebox[origin=c]{22}{o}}}_{j} } \left( { - ln\left( {e^{{ - \left( {\left( {\sum\limits_{{i = 1}}^{n} {{\mathfrak{H}}_{i} } \left( { - ln\left( {1 - \left( {u_{{A_{o} }} } \right)^{q} } \right)} \right)^{\tau } } \right)^{{\frac{1}{\tau }}} } \right)}} } \right)} \right)^{\tau } } \right)^{{\frac{1}{\tau }}} }} }},e^{{ - \left( {\sum\limits_{{j = 1}}^{m} {{\calligra{\rotatebox[origin=c]{22}{o}}}_{j} } \left( { - ln\left( {e^{{ - \left( {\sum\limits_{{i = 1}}^{n} {{\mathfrak{H}}_{i} } \left( { - ln\left( {v_{{A_{o} }} } \right)} \right)^{\tau } } \right)^{{\frac{1}{\tau }}} }} } \right)} \right)^{\tau } } \right)^{{\frac{1}{\tau }}} }} } \\ \end{array} } \right) \\ \end{aligned}$$

As we know that $\sum_{i=1}^{n}{\mathfrak{H}}_{i}=1$ and $\sum_{j=1}^{m}{\calligra{\rotatebox[origin=c]{22}{o}}}_{j}=1$, so

$$q-ROFSAAWA\left({\beth }_{{A}_{11}},{\beth }_{{A}_{12}},\dots \dots {\beth }_{{A}_{nm}}\right)=\left(\begin{array}{c}\sqrt[q]{1-{e}^{-{\left({\left(-ln\left({e}^{-\left({\left({\left(-ln\left(1-{\left({u}_{{A}_{o}}\right)}^{q}\right)\right)}^{\tau }\right)}^{\frac{1}{\tau }}\right)}\right)\right)}^{\tau }\right)}^{\frac{1}{\tau }}}}, {e}^{-{\left({\left(-ln\left({e}^{-{\left({\left(-ln\left({v}_{{A}_{o}}\right)\right)}^{\tau }\right)}^{\frac{1}{\tau }}}\right)\right)}^{\tau }\right)}^{\frac{1}{\tau }}}\end{array}\right)$$

$$=\left(\begin{array}{c}\sqrt[q]{1-{e}^{-\left(-ln\left({e}^{-\left(-ln\left(1-{\left({u}_{{A}_{o}}\right)}^{q}\right)\right)}\right)\right)}}, {e}^{-\left(-ln\left({e}^{-\left(-ln\left({v}_{{A}_{o}}\right)\right)}\right)\right)}\end{array}\right)$$

$$=\left(\begin{array}{c}\sqrt[q]{1-{e}^{ln\left({e}^{ln\left(1-{\left({u}_{{A}_{o}}\right)}^{q}\right)}\right)}}, {e}^{ln\left({v}_{{A}_{o}}\right)}\end{array}\right)$$

$$= \left( {\begin{array}{*{20}c} {\sqrt[q]{{1 - \left( {1 - \left( {u_{{A_{o} }} } \right)^{q} } \right)}}, v_{{A_{o} }} } \\ \end{array} } \right) = \left( {\begin{array}{*{20}c} {u_{{A_{o} }} v_{{A_{o} }} } \\ \end{array} } \right).$$

### Boundedness

Let ${\beth }_{{A}_{ij}}=\left({u}_{{A}_{ij}}, {v}_{{A}_{ij}}\right)$ be a collection of q-ROFSVs such that ${\beth }_{{A}_{ij}}^{-}=min\left({\beth }_{{A}_{11}}, {\beth }_{{A}_{12}},\dots , {\beth }_{{A}_{ij}}\right)$ and ${\beth }_{{A}_{ij}}^{+}=max\left({\beth }_{{A}_{11}}, {\beth }_{{A}_{12}},\dots , {\beth }_{{A}_{ij}}\right)$. Then

$${\beth }_{{A}_{ij}}^{-}\le q-ROFSAAWA\left({\beth }_{{A}_{11}},{\beth }_{{A}_{12}},\dots \dots {\beth }_{{A}_{nm}}\right)\le {\beth }_{{A}_{ij}}^{+}$$

#### Proof

Let ${\beth }_{A}^{-}=min\left\{{\beth }_{{A}_{11}}, {\beth }_{{A}_{12}},\dots ,{\beth }_{{A}_{ij}}\right\}=\left({u}_{{A}_{ij}}^{-},{v}_{{A}_{ij}}^{-}\right)$ and ${\beth }_{A}^{+}=max\left\{{\beth }_{{A}_{11}}, {\beth }_{{A}_{12}},\dots ,{\beth }_{{A}_{ij}}\right\}=\left({u}_{{A}_{ij}}^{+},{v}_{{A}_{ij}}^{+}\right)$. We have ${u}_{{A}_{ij}}^{-}=min\left\{{u}_{{A}_{ij}}\right\}$, ${v}_{{A}_{ij}}^{-}=max\left\{{v}_{{A}_{ij}}\right\}$, ${u}_{{A}_{ij}}^{+}=max\left\{{v}_{{A}_{ij}}\right\}$, and ${v}_{{A}_{ij}}^{+}=min\left\{{v}_{{A}_{ij}}\right\}$. Then, the obtained aggregated value of the q-ROFSAAWA operator should satisfy the

$$\begin{aligned} & \sqrt[q]{{1 - e^{{ - \left( {\sum\limits_{{j = 1}}^{m} {{\calligra{\rotatebox[origin=c]{22}{o}}}_{j} } \left( { - ln\left( {e^{{ - \left( {\sum\limits_{{i = 1}}^{n} {{\mathfrak{H}}_{i} } \left( { - ln\left( {1 - \left( {u_{{A_{{ij}} }}^{ - } } \right)^{q} } \right)} \right)^{\tau } } \right)^{{\frac{1}{\tau }}} }} } \right)} \right)^{\tau } } \right)^{{\frac{1}{\tau }}} }} }} \\ & \quad \le \sqrt[q]{{1 - e^{{ - \left( {\sum\limits_{{j = 1}}^{m} {{\calligra{\rotatebox[origin=c]{22}{o}}}_{j} } \left( { - ln\left( {e^{{ - \left( {\sum\limits_{{i = 1}}^{n} {{\mathfrak{H}}_{i} } \left( { - ln\left( {1 - \left( {u_{{A_{{ij}} }} } \right)^{q} } \right)} \right)^{\tau } } \right)^{{\frac{1}{\tau }}} }} } \right)} \right)^{\tau } } \right)^{{\frac{1}{\tau }}} }} }} \le \sqrt[q]{{1 - e^{{ - \left( {\sum\limits_{{j = 1}}^{m} {{\calligra{\rotatebox[origin=c]{22}{o}}}_{j} } \left( { - ln\left( {e^{{ - \left( {\sum\limits_{{i = 1}}^{n} {{\mathfrak{H}}_{i} } \left( { - ln\left( {1 - \left( {u_{{A_{{ij}} }}^{ + } } \right)^{q} } \right)} \right)^{\tau } } \right)^{{\frac{1}{\tau }}} }} } \right)} \right)^{\tau } } \right)^{{\frac{1}{\tau }}} }} }} \\ \end{aligned}$$

Which can be verified as follows:

As $${u}_{{A}_{ij}}^{-}$$$$\le {u}_{{A}_{ij}}$$$$\le {u}_{{A}_{ij}}^{+}$$ $$\Rightarrow$$$${({u}_{{A}_{ij}}^{-})}^{q}$$ $$\le$$$${({u}_{{A}_{ij}})}^{q}$$$$\le$$$${({u}_{{A}_{ij}}^{+})}^{q}$$$${({u}_{{A}_{ij}}^{-})}^{q}$$$$\le$$$${({u}_{{A}_{ij}})}^{q}$$$$\le$$ $${({u}_{{A}_{ij}}^{+})}^{q}$$$$\Rightarrow 1-$$$${({u}_{{A}_{ij}}^{-})}^{q}$$ $$\ge 1-$$$${({u}_{{A}_{ij}})}^{q}$$$$\ge 1-$$$${({u}_{{A}_{ij}}^{+})}^{q}$$ $$\Rightarrow ln(1-$$$${({u}_{{A}_{ij}}^{-})}^{q})$$ $$\ge ln(1-$$$${({u}_{{A}_{ij}})}^{q})$$$$\ge ln(1-$$$${({u}_{{A}_{ij}}^{+})}^{q})$$ $$\Rightarrow -ln(1-$$$${({u}_{{A}_{ij}}^{-})}^{q})$$ $$\le -ln(1-$$$${({u}_{{A}_{ij}})}^{q})$$$$\le -ln(1-$$$${({u}_{{A}_{ij}}^{+})}^{q})$$ $$\Rightarrow$$$${(-ln(1-{({u}_{{A}_{ij}}^{-})}^{q}))}^{\tau }$$ $$\le$$$${(-ln(1-{({u}_{{A}_{ij}})}^{q}))}^{\tau }$$ $$\le$$$${(-ln(1-{({u}_{{A}_{ij}}^{+})}^{q}))}^{\tau }$$ $$\Rightarrow$$$$\sum_{i=1}^{n}{\mathfrak{H}}_{i}$$ $${(-ln(1-{({u}_{{A}_{ij}}^{-})}^{q}))}^{\tau }$$ $$\le$$$$\sum_{i=1}^{n}{\mathfrak{H}}_{i}$$ $${(-ln(1-{({u}_{{A}_{ij}})}^{q}))}^{\tau }$$ $$\le$$$$\sum_{i=1}^{n}$$$${\mathfrak{H}}_{i}$$ $${(-ln(1-{({u}_{{A}_{ij}}^{+})}^{q}))}^{\tau }$$ $$\Rightarrow$$$$(\sum_{i=1}^{n}{\mathfrak{H}}_{i}$$$${(-ln(1-{({u}_{{A}_{ij}}^{-})}^{q}))}^{\tau })^{\frac{1}{\tau }}$$ $$\le$$ $$(\sum\nolimits_{{i = 1}}^{n} {{\mathfrak{H}}_{i} } ( - ln$$$$(1 - (u_{{A_{{ij}} }} )^{q} ))^{\tau } )^{{\frac{1}{\tau }}}$$ $$\le$$ $$(\sum\nolimits_{{i = 1}}^{n} {{\mathfrak{H}}_{i} } ( - ln$$$$(1 - (u_{{A_{{ij}} }}^{ + } )^{q} ))^{\tau } )^{{\frac{1}{\tau }}}$$ $$\Rightarrow$$$$- (\sum\nolimits_{{i = 1}}^{n} {{\mathfrak{H}}_{i} } ( - ln$$$$(1 - (u_{{A_{{ij}} }}^{ - } )^{q} ))^{\tau } )^{{\frac{1}{\tau }}}$$ $$\ge$$$$- (\sum\nolimits_{{i = 1}}^{n} {{\mathfrak{H}}_{i} } ( - ln(1 -$$$$(u_{{A_{{ij}} }} )^{q} ))^{\tau } )^{{\frac{1}{\tau }}}$$$$\ge$$$$- (\sum\nolimits_{{i = 1}}^{n} {{\mathfrak{H}}_{i} } ( - ln(1 -$$$$(u_{{A_{{ij}} }}^{ + } )^{q} ))^{\tau } )^{{\frac{1}{\tau }}}$$ $$\Rightarrow {e}^{-{(\sum_{i=1}^{n}{\mathfrak{H}}_{i}{(-ln(1-{({u}_{{A}_{ij}}^{-})}^{q}))}^{\tau })}^{\frac{1}{\tau }}}$$$$e^{{ - (\sum\nolimits_{{i = 1}}^{n} {{\mathfrak{H}}_{i} } ( - ln(1 - }}$$$$(u_{{A_{{ij}} }}^{ - } )^{q} ))^{\tau } )^{{\frac{1}{\tau }}}$$ $$\ge$$ $$e^{{ - (\sum\nolimits_{{i = 1}}^{n} {{\mathfrak{H}}_{i} } ( - ln(}}$$ $$1 - (u_{{A_{{ij}} }} )^{q} ))^{\tau } )^{{\frac{1}{\tau }}}$$$$\ge$$ $$e^{{ - (\sum\nolimits_{{i = 1}}^{n} {{\mathfrak{H}}_{i} } ( - ln(1 - }}$$ $$(u_{{A_{{ij}} }}^{ + } )^{q} ))^{\tau } )^{{\frac{1}{\tau }}}$$ $$\Rightarrow$$$$-ln({e}^{-{(\sum_{i=1}^{n}{\mathfrak{H}}_{i}{(-ln(1-{({u}_{{A}_{ij}}^{-})}^{q}))}^{\tau })}^{\frac{1}{\tau }}})$$ $$\le -ln({e}^{-{(\sum_{i=1}^{n}{\mathfrak{H}}_{i}{(-ln(1-{({u}_{{A}_{ij}})}^{q}))}^{\tau })}^{\frac{1}{\tau }}})$$$$\le -ln({e}^{-{(\sum_{i=1}^{n}{\mathfrak{H}}_{i}{(-ln(1-{({u}_{{A}_{ij}}^{+})}^{q}))}^{\tau })}^{\frac{1}{\tau }}})$$ $$\sum_{j=1}^{m}{\calligra{\rotatebox[origin=c]{22}{o}}}_{j}{(-ln({e}^{-{(\sum_{i=1}^{n}{\mathfrak{H}}_{i} {(-ln(1-{({u}_{{A}_{ij}}^{-})}^{q}))}^{\tau })}^{\frac{1}{\tau }}}))}^{\tau }$$$$\Rightarrow \le \sum_{j=1}^{m}{\calligra{\rotatebox[origin=c]{22}{o}}}_{j}{(-ln({e}^{-{(\sum_{i=1}^{n}{\mathfrak{H}}_{i}{(-ln(1-{({u}_{{A}_{ij}})}^{q}))}^{\tau })}^{\frac{1}{\tau }}}))}^{\tau }$$$$\le \sum_{j=1}^{m}{\calligra{\rotatebox[origin=c]{22}{o}}}_{j}{(-ln({e}^{-{(\sum_{i=1}^{n}{\mathfrak{H}}_{i}{(-ln(1-{({u}_{{A}_{ij}}^{+})}^{q}))}^{\tau })}^{\frac{1}{\tau }}}))}^{\tau }$$$$\Rightarrow {-(\sum_{j=1}^{m}{\calligra{\rotatebox[origin=c]{22}{o}}}_{j}{(-ln({e}^{-{(\sum_{i=1}^{n}{\mathfrak{H}}_{i}{(-ln(1-{({u}_{{A}_{ij}}^{-})}^{q}))}^{\tau })}^{\frac{1}{\tau }}}))}^{\tau })}^{\frac{1}{\tau }}$$$$\ge {-(\sum_{j=1}^{m}{\calligra{\rotatebox[origin=c]{22}{o}}}_{j}{(-ln({e}^{-{(\sum_{i=1}^{n}{\mathfrak{H}}_{i}{(-ln(1-{({u}_{{A}_{ij}})}^{q}))}^{\tau })}^{\frac{1}{\tau }}}))}^{\tau })}^{\frac{1}{\tau }}$$$$\ge {-(\sum_{j=1}^{m}{\calligra{\rotatebox[origin=c]{22}{o}}}_{j}{(-ln({e}^{-{(\sum_{i=1}^{n}{\mathfrak{H}}_{i}{(-ln(1-{({u}_{{A}_{ij}}^{+})}^{q}))}^{\tau })}^{\frac{1}{\tau }}}))}^{\tau })}^{\frac{1}{\tau }}$$$$\Rightarrow {e}^{{-(\sum_{j=1}^{m}{\calligra{\rotatebox[origin=c]{22}{o}}}_{j}{(-ln({e}^{-{(\sum_{i=1}^{n}{\mathfrak{H}}_{i}{(-ln(1-{({u}_{{A}_{ij}}^{-})}^{q}))}^{\tau })}^{\frac{1}{\tau }}}))}^{\tau })}^{\frac{1}{\tau }}}$$$$\ge {e}^{{-(\sum_{j=1}^{m}{\calligra{\rotatebox[origin=c]{22}{o}}}_{j}{(-ln({e}^{-{(\sum_{i=1}^{n}{\mathfrak{H}}_{i}{(-ln(1-{({u}_{{A}_{ij}})}^{q}))}^{\tau })}^{\frac{1}{\tau }}}))}^{\tau })}^{\frac{1}{\tau }}}$$$$\ge {e}^{{-(\sum_{j=1}^{m}{\calligra{\rotatebox[origin=c]{22}{o}}}_{j}{(-ln({e}^{-{(\sum_{i=1}^{n}{\mathfrak{H}}_{i}{(-ln(1-{({u}_{{A}_{ij}}^{+})}^{q}))}^{\tau })}^{\frac{1}{\tau }}}))}^{\tau })}^{\frac{1}{\tau }}}$$$$\Rightarrow 1-{e}^{{-(\sum_{j=1}^{m}{\calligra{\rotatebox[origin=c]{22}{o}}}_{j}{(-ln({e}^{-{(\sum_{i=1}^{n}{\mathfrak{H}}_{i}{(-ln(1-{({u}_{{A}_{ij}}^{-})}^{q}))}^{\tau })}^{\frac{1}{\tau }}}))}^{\tau })}^{\frac{1}{\tau }}}$$$$\le 1-{e}^{{-(\sum_{j=1}^{m}{\calligra{\rotatebox[origin=c]{22}{o}}}_{j}{(-ln({e}^{-{(\sum_{i=1}^{n}{\mathfrak{H}}_{i}{(-ln(1-{({u}_{{A}_{ij}})}^{q}))}^{\tau })}^{\frac{1}{\tau }}}))}^{\tau })}^{\frac{1}{\tau }}}$$$$\le 1-{e}^{{-(\sum_{j=1}^{m}{\calligra{\rotatebox[origin=c]{22}{o}}}_{j}{(-ln({e}^{-{(\sum_{i=1}^{n}{\mathfrak{H}}_{i}{(-ln(1-{({u}_{{A}_{ij}}^{+})}^{q}))}^{\tau })}^{\frac{1}{\tau }}}))}^{\tau })}^{\frac{1}{\tau }}}$$$$\Rightarrow \sqrt[q]{1-{e}^{{-(\sum_{j=1}^{m}{\calligra{\rotatebox[origin=c]{22}{o}}}_{j}{(-ln({e}^{-{(\sum_{i=1}^{n}{\mathfrak{H}}_{i}{(-ln(1-{({u}_{{A}_{ij}}^{-})}^{q}))}^{\tau })}^{\frac{1}{\tau }}}))}^{\tau })}^{\frac{1}{\tau }}}}$$$$\le \sqrt[q]{1-{e}^{{-(\sum_{j=1}^{m}{\calligra{\rotatebox[origin=c]{22}{o}}}_{j}{(-ln({e}^{-{(\sum_{i=1}^{n}{\mathfrak{H}}_{i}{(-ln(1-{({u}_{{A}_{ij}})}^{q}))}^{\tau })}^{\frac{1}{\tau }}}))}^{\tau })}^{\frac{1}{\tau }}}}$$$$\le \sqrt[q]{1-{e}^{{-(\sum_{j=1}^{m}{\calligra{\rotatebox[origin=c]{22}{o}}}_{j}{(-ln({e}^{-{(\sum_{i=1}^{n}{\mathfrak{H}}_{i}{(-ln(1-{({u}_{{A}_{ij}}^{+})}^{q}))}^{\tau })}^{\frac{1}{\tau }}}))}^{\tau })}^{\frac{1}{\tau }}}}$$

Hence,

$${\beth }_{{A}_{ij}}^{-}\le q-ROFSAAWA\left({\beth }_{{A}_{11}},{\beth }_{{A}_{12}},\dots \dots {\beth }_{{A}_{nm}}\right)\le {\beth }_{{A}_{ij}}^{+}$$

#### Monotonicity

Let ${\beth }_{{A}_{ij}}=\left({u}_{{A}_{ij}}, {v}_{{A}_{ij}}\right)$ and ${\beth }_{{A}_{ij}}^{*} =\left({u}_{{A}_{ij}}^{*}, {v}_{{A}_{ij}}^{*}\right)$ be two distinct families of q-ROFSVs. Then $q-ROFSAAWA\left({\beth }_{{A}_{11}},{\beth }_{{A}_{12}},\dots \dots {\beth }_{{A}_{nm}}\right)\le q-ROFSAAWA \left({\beth }_{{A}_{11}}^{*},{\beth }_{{A}_{12}}^{*},\dots \dots \dots , {\beth }_{{A}_{nm}}^{*}\right)$, if ${\beth }_{{A}_{ij}}\le {\beth }_{{A}_{ij}}^{*}$ $\forall i, j$.

##### Proof

As $${\beth }_{{A}_{ij}}=\left({u}_{{A}_{ij}}, {v}_{{A}_{ij}}\right)$$ and $${\beth }_{{A}_{ij}}^{*} =\left({u}_{{A}_{ij}}^{*}, {v}_{{A}_{ij}}^{*}\right)$$ be two distinct collections of q-ROFSVs, such as $${\beth }_{{A}_{ij}}\le {\beth }_{{A}_{ij}}^{*}$$. Then,$${u}_{{A}_{ij}}\le {u}_{{A}_{ij}}^{*}\Rightarrow {\left({u}_{{A}_{ij}}\right)}^{q}\le {\left({u}_{{A}_{ij}}^{*}\right)}^{q}\Rightarrow 1-{\left({u}_{{A}_{ij}}\right)}^{q}\ge 1-{\left({u}_{{A}_{ij}}^{*}\right)}^{q}$$$$\Rightarrow ln\left(1-{\left({u}_{{A}_{ij}}\right)}^{q}\right)\ge ln\left(1-{\left({u}_{{A}_{ij}}^{*}\right)}^{q}\right)\Rightarrow -ln\left(1-{\left({u}_{{A}_{ij}}\right)}^{q}\right)\le -ln\left(1-{\left({u}_{{A}_{ij}}^{*}\right)}^{q}\right)$$$$\Rightarrow {\left(-ln\left(1-{\left({u}_{{A}_{ij}}\right)}^{q}\right)\right)}^{\tau }\le {\left(-ln\left(1-{\left({u}_{{A}_{ij}}^{*}\right)}^{q}\right)\right)}^{\tau }$$$$\Rightarrow \sum_{i=1}^{n}{\mathfrak{H}}_{i}{\left(-ln\left(1-{\left({u}_{{A}_{ij}}\right)}^{q}\right)\right)}^{\tau }\le \sum_{i=1}^{n}{\mathfrak{H}}_{i}{\left(-ln\left(1-{\left({u}_{{A}_{ij}}^{*}\right)}^{q}\right)\right)}^{\tau }$$$$\Rightarrow {\left(\sum_{i=1}^{n}{\mathfrak{H}}_{i}{\left(-ln\left(1-{\left({u}_{{A}_{ij}}\right)}^{q}\right)\right)}^{\tau }\right)}^{\frac{1}{\tau }}\le {\left(\sum_{i=1}^{n}{\mathfrak{H}}_{i}{\left(-ln\left(1-{\left({u}_{{A}_{ij}}^{*}\right)}^{q}\right)\right)}^{\tau }\right)}^{\frac{1}{\tau }}$$$$\Rightarrow -{\left(\sum_{i=1}^{n}{\mathfrak{H}}_{i}{\left(-ln\left(1-{\left({u}_{{A}_{ij}}\right)}^{q}\right)\right)}^{\tau }\right)}^{\frac{1}{\tau }}\ge -{\left(\sum_{i=1}^{n}{\mathfrak{H}}_{i}{\left(-ln\left(1-{\left({u}_{{A}_{ij}}^{*}\right)}^{q}\right)\right)}^{\tau }\right)}^{\frac{1}{\tau }}$$$$\Rightarrow -{\left(\sum_{i=1}^{n}{\mathfrak{H}}_{i}{\left(-ln\left(1-{\left({u}_{{A}_{ij}}\right)}^{q}\right)\right)}^{\tau }\right)}^{\frac{1}{\tau }}\ge -{\left(\sum_{i=1}^{n}{\mathfrak{H}}_{i}{\left(-ln\left(1-{\left({u}_{{A}_{ij}}^{*}\right)}^{q}\right)\right)}^{\tau }\right)}^{\frac{1}{\tau }}$$$$\Rightarrow {e}^{-{\left(\sum_{i=1}^{n}{\mathfrak{H}}_{i}{\left(-ln\left(1-{\left({u}_{{A}_{ij}}\right)}^{q}\right)\right)}^{\tau }\right)}^{\frac{1}{\tau }}}\ge {e}^{-{\left(\sum_{i=1}^{n}{\mathfrak{H}}_{i}{\left(-ln\left(1-{\left({u}_{{A}_{ij}}^{*}\right)}^{q}\right)\right)}^{\tau }\right)}^{\frac{1}{\tau }}}$$$$\Rightarrow -ln\left({e}^{-{\left(\sum_{i=1}^{n}{\mathfrak{H}}_{i}{\left(-ln\left(1-{\left({u}_{{A}_{ij}}\right)}^{q}\right)\right)}^{\tau }\right)}^{\frac{1}{\tau }}}\right)\le -ln\left({e}^{-{\left(\sum_{i=1}^{n}{\mathfrak{H}}_{i}{\left(-ln\left(1-{\left({u}_{{A}_{ij}}^{*}\right)}^{q}\right)\right)}^{\tau }\right)}^{\frac{1}{\tau }}}\right)$$$$\Rightarrow {-\left(\sum_{j=1}^{m}{\calligra{\rotatebox[origin=c]{22}{o}}}_{j}{\left(-ln\left({e}^{-{\left(\sum_{i=1}^{n}{\mathfrak{H}}_{i}{\left(-ln\left(1-{\left({u}_{{A}_{ij}}\right)}^{q}\right)\right)}^{\tau }\right)}^{\frac{1}{\tau }}}\right)\right)}^{\tau }\right)}^{\frac{1}{\tau }}\ge {-\left(\sum_{j=1}^{m}{\calligra{\rotatebox[origin=c]{22}{o}}}_{j}{\left(-ln\left({e}^{-{\left(\sum_{i=1}^{n}{\mathfrak{H}}_{i}{\left(-ln\left(1-{\left({u}_{{A}_{ij}}^{*}\right)}^{q}\right)\right)}^{\tau }\right)}^{\frac{1}{\tau }}}\right)\right)}^{\tau }\right)}^{\frac{1}{\tau }}$$$${\Rightarrow e}^{{-\left(\sum_{j=1}^{m}{\calligra{\rotatebox[origin=c]{22}{o}}}_{j}{\left(-ln\left({e}^{-{\left(\sum_{i=1}^{n}{\mathfrak{H}}_{i}{\left(-ln\left(1-{\left({u}_{{A}_{ij}}\right)}^{q}\right)\right)}^{\tau }\right)}^{\frac{1}{\tau }}}\right)\right)}^{\tau }\right)}^{\frac{1}{\tau }}}\ge {e}^{{-\left(\sum_{j=1}^{m}{\calligra{\rotatebox[origin=c]{22}{o}}}_{j}{\left(-ln\left({e}^{-{\left(\sum_{i=1}^{n}{\mathfrak{H}}_{i}{\left(-ln\left(1-{\left({u}_{{A}_{ij}}^{*}\right)}^{q}\right)\right)}^{\tau }\right)}^{\frac{1}{\tau }}}\right)\right)}^{\tau }\right)}^{\frac{1}{\tau }}}$$$$\Rightarrow 1-{e}^{{-\left(\sum_{j=1}^{m}{\calligra{\rotatebox[origin=c]{22}{o}}}_{j}{\left(-ln\left({e}^{-{\left(\sum_{i=1}^{n}{\mathfrak{H}}_{i}{\left(-ln\left(1-{\left({u}_{{A}_{ij}}\right)}^{q}\right)\right)}^{\tau }\right)}^{\frac{1}{\tau }}}\right)\right)}^{\tau }\right)}^{\frac{1}{\tau }}}\le 1-{e}^{{-\left(\sum_{j=1}^{m}{\calligra{\rotatebox[origin=c]{22}{o}}}_{j}{\left(-ln\left({e}^{-{\left(\sum_{i=1}^{n}{\mathfrak{H}}_{i}{\left(-ln\left(1-{\left({u}_{{A}_{ij}}^{*}\right)}^{q}\right)\right)}^{\tau }\right)}^{\frac{1}{\tau }}}\right)\right)}^{\tau }\right)}^{\frac{1}{\tau }}}$$$$\Rightarrow \sqrt[q]{1-{e}^{{-\left(\sum_{j=1}^{m}{\calligra{\rotatebox[origin=c]{22}{o}}}_{j}{\left(-ln\left({e}^{-{\left(\sum_{i=1}^{n}{\mathfrak{H}}_{i}{\left(-ln\left(1-{\left({u}_{{A}_{ij}}\right)}^{q}\right)\right)}^{\tau }\right)}^{\frac{1}{\tau }}}\right)\right)}^{\tau }\right)}^{\frac{1}{\tau }}}}\le \sqrt[q]{1-{e}^{{-\left(\sum_{j=1}^{m}{\calligra{\rotatebox[origin=c]{22}{o}}}_{j}{\left(-ln\left({e}^{-{\left(\sum_{i=1}^{n}{\mathfrak{H}}_{i}{\left(-ln\left(1-{\left({u}_{{A}_{ij}}^{*}\right)}^{q}\right)\right)}^{\tau }\right)}^{\frac{1}{\tau }}}\right)\right)}^{\tau }\right)}^{\frac{1}{\tau }}}}$$$$\Rightarrow u_{{A_{ij} }} \le u_{{A_{ij} }}^{*} ,$$

##### Definition 15

Let ${\beth }_{{A}_{ij}}=\left({u}_{{A}_{ij}}, {v}_{{A}_{ij}}\right)$ be a collection of q-ROFSVs, where $i,j=\text{1,2},\dots \dots .n,m$. Then, a q-ROFSAAWG is defined as:

$$q-ROFSAAWG\left({\beth }_{{A}_{11}},{\beth }_{{A}_{12}},\dots \dots {\beth }_{{A}_{nm}}\right)={\calligra{\rotatebox[origin=c]{22}{o}}}_{1}{\mathfrak{H}}_{1}{\beth }_{{A}_{11}}\otimes{\calligra{\rotatebox[origin=c]{22}{o}}}_{1}{\mathfrak{H}}_{2}{\beth }_{{A}_{12}}\otimes\dots \dots \otimes{\calligra{\rotatebox[origin=c]{22}{o}}}_{m}{\mathfrak{H}}_{n}{\beth }_{{A}_{nm}}={\left(\stackrel{m}{\underset{j=1}{\otimes}}{\left(\stackrel{n}{\underset{i=1}{\otimes}}{\beth }_{{A}_{ij}}\right)}^{{\mathfrak{H}}_{i}}\right)}^{{\calligra{\rotatebox[origin=c]{22}{o}}}_{j}}$$

where, ${\mathfrak{H}}_{i}$ and ${\calligra{\rotatebox[origin=c]{22}{o}}}_{j}$ be the weights of experts and attributes such as ${\mathfrak{H}}_{i}>0,\sum_{i=1}^{n}{\mathfrak{H}}_{i}=1$ and ${\calligra{\rotatebox[origin=c]{22}{o}}}_{j}>0,\sum_{j=1}^{m}{\calligra{\rotatebox[origin=c]{22}{o}}}_{j}=1$.

##### Theorem 3

*Let* ${\beth }_{{A}_{ij}}=\left({u}_{{A}_{ij}}, {v}_{{A}_{ij}}\right)$ *be a collection of q-ROFSVs where* $i,j=\text{1,2},\dots \dots .n,m$. *Then, the aggregated outcome of the q-ROFSAAWG operator is also a q-ROFSV and*

3 $$\begin{aligned} & q - ROFSAAWG\left( {{\beth }_{{A_{{11}} }} ,{\beth }_{{A_{{12}} }} , \ldots \ldots {\beth }_{{A_{{nm}} }} } \right) \\ & \quad = \left( {\begin{array}{*{20}c} {e^{{ - \left( {\sum\limits_{{j = 1}}^{m} {\calligra{\rotatebox[origin=c]{22}{o}}}_{j} \left( { - ln\left( {e^{{ - \left( {\sum\limits_{{i = 1}}^{n} {{\mathfrak{H}}_{i} } \left( { - ln\left( {u_{{A_{{ij}} }} } \right)} \right)^{\tau } } \right)^{{\frac{1}{\tau }}} }} } \right)} \right)^{\tau } } \right)^{{\frac{1}{\tau }}} }} ,\sqrt {1 - e^{{ - \left( {\sum\limits_{{j = 1}}^{m} {\calligra{\rotatebox[origin=c]{22}{o}}}_{j} \left( { - ln\left( {e^{{ - \left( {\left( {\sum\limits_{{i = 1}}^{n} {{\mathfrak{H}}_{i} } \left( { - ln\left( {1 - \left( {v_{{A_{{ij}} }} } \right)^{q} } \right)} \right)^{\tau } } \right)^{{\frac{1}{\tau }}} } \right)}} } \right)} \right)^{\tau } } \right)^{{\frac{1}{\tau }}} }} ,} } \\ \end{array} } \right) \\ \end{aligned}$$

*where* ${\mathfrak{H}}_{i}$ *and* ${\calligra{\rotatebox[origin=c]{22}{o}}}_{j}$ *be the weights of experts and attributes such as* ${\mathfrak{H}}_{i}>0,\sum_{i=1}^{n}{\mathfrak{H}}_{i}=1$ *and* ${\calligra{\rotatebox[origin=c]{22}{o}}}_{j}>0,\sum_{j=1}^{m}{\calligra{\rotatebox[origin=c]{22}{o}}}_{j}=1$.

Proof: Similar Theorem 2.

##### Proposition 2

*If* ${\beth }_{{A}_{ij}}=\left({u}_{{A}_{ij}}, {v}_{{A}_{ij}}\right)$; $\left(i=1, 2, 3, \dots , n;j=1, 2, 3, \dots ,m\right)$ *be a collection of q-ROFSVs and* ${\mathfrak{H}}_{i}$, ${\calligra{\rotatebox[origin=c]{22}{o}}}_{j}$ *be the weights of experts and attributes such as* ${\mathfrak{H}}_{i}>0,\sum_{i=1}^{n}{\mathfrak{H}}_{i}=1$ *and* ${\calligra{\rotatebox[origin=c]{22}{o}}}_{j}>0,\sum_{j=1}^{m}{\calligra{\rotatebox[origin=c]{22}{o}}}_{j}=1$.

#### Idempotency

Let ${\beth }_{{A}_{ij}}={\beth }_{{A}_{o}}=\left({u}_{{A}_{o}}, {v}_{{A}_{o}}\right)$ is satisfied for any $i=1, 2, 3, \dots , n;j=1, 2, 3, \dots ,m$. Then,

$$q-ROFSAAWG\left({\beth }_{{A}_{11}},{\beth }_{{A}_{12}},\dots \dots {\beth }_{{A}_{nm}}\right)={\beth }_{{A}_{o}}$$

#### Boundedness

Let ${\beth }_{{A}_{ij}}^{-}=\left(min\left({u}_{{A}_{ij}}\right), max\left({v}_{{A}_{ij}}\right)\right)$ and ${\beth }_{{A}_{ij}}^{+}=\left(max\left({u}_{{A}_{ij}}\right), min\left({v}_{{A}_{ij}}\right)\right)$. Then

$${\beth }_{{A}_{ij}}^{-}\le q-ROFSAAWG\left({\beth }_{{A}_{11}},{\beth }_{{A}_{12}},\dots \dots {\beth }_{{A}_{nm}}\right)\le {\beth }_{{A}_{ij}}^{+}$$

#### Monotonicity

Let ${\beth }_{{A}_{ij}}=\left({u}_{{A}_{ij}}, {v}_{{A}_{ij}}\right)$ and ${\beth }_{{A}_{ij}}^{*} =\left({u}_{{A}_{ij}}^{*}, {v}_{{A}_{ij}}^{*}\right)$ be two distinct families of q-ROFSVs. Then $q - ROFSAAWG\left( {\beth_{{A_{11} }} ,\beth_{{A_{12} }} , \ldots \ldots \beth_{{A_{nm} }} } \right) \le q - ROFSAAWG \left( {\beth_{{A_{11} }}^{*} ,\beth_{{A_{12} }}^{*} , \ldots \ldots \ldots , \beth_{{A_{nm} }}^{*} } \right),$

if $$\beth_{{A_{ij} }} \le \beth_{{A_{ij} }}^{*}$$ $$\forall { }i, j$$.

##### Proof

Similar to Proposition 1.

## MAGDM model in q-rung orthopair fuzzy soft structure using Aczel–Alsina operators

In multiple real-world scenarios, experts in decision-making may show contradicting opinions because of their different backgrounds, experiences, and assessment techniques. Decision-makers also often indicate their opinions using q-ROFSNs that reasonably reflect indeterminacy and confusion. The study we will conduct constitutes a novel MAGDM methodology that integrates q-ROFSVs based on our developed Aczel–Alsina AOs to overcome challenging decision-making issues. This technique aims to boost the governance of undetermined and conflicting aspects of decision-making procedures. Let ${\mathcal{S}}=\left\{{{\mathcal{S}}}_{1},{{\mathcal{S}}}_{2},\dots \dots {{\mathcal{S}}}_{k}\right\}$ and ${\mathcal{Z}}=\left\{{{\mathcal{Z}}}_{1},{{\mathcal{Z}}}_{2},\dots \dots {{\mathcal{Z}}}_{s}\right\}$ be a collection of $k$ alternatives and $s$ experts, respectively, and $\mathfrak{H}={\left({\mathfrak{H}}_{1}, {\mathfrak{H}}_{2},\dots , {\mathfrak{H}}_{n}\right)}^{T}$ be the weights of experts such as ${\mathfrak{H}}_{i}>0$ and $\sum_{i=1}^{n}{\mathfrak{H}}_{i}=1$. Let $\mathfrak{I}=\left\{{\mathfrak{I}}_{1},{\mathfrak{I}}_{2},\dots \dots {\mathfrak{I}}_{z}\right\}$ be a set of parameters with weights $\calligra{\rotatebox[origin=c]{22}{o}}={\left({\calligra{\rotatebox[origin=c]{22}{o}}}_{1}, {\calligra{\rotatebox[origin=c]{22}{o}}}_{2},\dots , {\calligra{\rotatebox[origin=c]{22}{o}}}_{n}\right)}^{T}$ such as ${\calligra{\rotatebox[origin=c]{22}{o}}}_{j}>0$ and $\sum_{j=1}^{m}{\calligra{\rotatebox[origin=c]{22}{o}}}_{j}=1$. The group of specialists delivers their opinions for each alternative in the type of q-ROFSNs, organized as ${\Delta }_{{A}_{ij}}^{p}=\left({u}_{{A}_{ij}}^{(p)}, {v}_{{A}_{ij}}^{(p)}\right)$, where $0\le {u}_{{A}_{ij}}^{(p)}, {v}_{{A}_{ij}}^{(p)}\le 1$ and ${\left({u}_{{A}_{ij}}^{(p)}\right)}^{2}+{\left({v}_{{A}_{ij}}^{(p)}\right)}^{2}\le 1$, $\forall i, j$.

The operations requisite to implementing the recommended method are listed in the subsequent order:

Step 1: Develop decision matrices for every alternative $\left\{{{\mathcal{S}}}_{z}: z = 1, 2, \dots , k\right\}$ by using the available factors communicated as q-ROFSNs.

$${\left[{\Delta }_{{A}_{ij}}^{p}\right]}_{n\times m}=\begin{array}{c}{{\mathcal{Z}}}_{1}\\ {{\mathcal{Z}}}_{2}\\ \vdots \\ {{\mathcal{Z}}}_{n}\end{array}\left(\begin{array}{cccc}\left({u}_{{A}_{11}}, {v}_{{A}_{11}}\right)& \left({u}_{{A}_{12}}, {v}_{{A}_{12}}\right)& \cdots & \left({u}_{{A}_{1m}}, {v}_{{A}_{1m}}\right)\\ \left({u}_{{A}_{21}}, {v}_{{A}_{21}}\right)& \left({u}_{{A}_{22}}, {v}_{{A}_{22}}\right)& \cdots & \left({u}_{{A}_{2m}}, {v}_{{A}_{2m}}\right)\\ \vdots & \vdots & \vdots & \vdots \\ \left({u}_{{A}_{n1}}, {v}_{{A}_{n1}}\right)& \left({u}_{{A}_{n2}}, {v}_{{A}_{n2}}\right)& \cdots & \left({u}_{{A}_{nm}}, {v}_{{A}_{nm}}\right)\end{array}\right)$$

Step 2: To obtain the matrix ${\left[{\Delta }_{{A}_{ij}}^{p}\right]}_{n\times m}$, the factors are divided into two types: cost-related and benefit-related. The normalization process is not needed when every factor in the data matrix is identical. When the decision matrix involves multiple aspects, including costs and benefits, normalization is necessary for regulating the information for instructive assessments. The normalizing rule stated in Eq. 4.1 is carried out for this particular phase.

4 $${\Phi }_{ij}^{(z)}=\left\{\begin{array}{c}{\left({\Delta }_{{A}_{ij}}^{p}\right)}^{c}= \left({v}_{{A}_{ij}}, {u}_{{A}_{ij}}\right)\\ {\Delta }_{{A}_{ij}}^{p}= \left({u}_{{A}_{ij}}, {v}_{{A}_{ij}}\right)\end{array}\right.$$

Step 3: Apply Eqs. 3.1 or 3.2 to calculate the aggregated decision matrices for every alternative ${\mathcal{S}}=\left\{{{\mathcal{S}}}_{1},{{\mathcal{S}}}_{2},\dots \dots {{\mathcal{S}}}_{k}\right\}$.

Step 4: Evaluate the score/accuracy values for every alternate using Eq. 2.1.

Step 5: Locate the most appropriate alternative built on the most prevalent score/accuracy value.

Step 6: Schedule the alternates in order of their score/accuracy values.

**Fig. 2:**
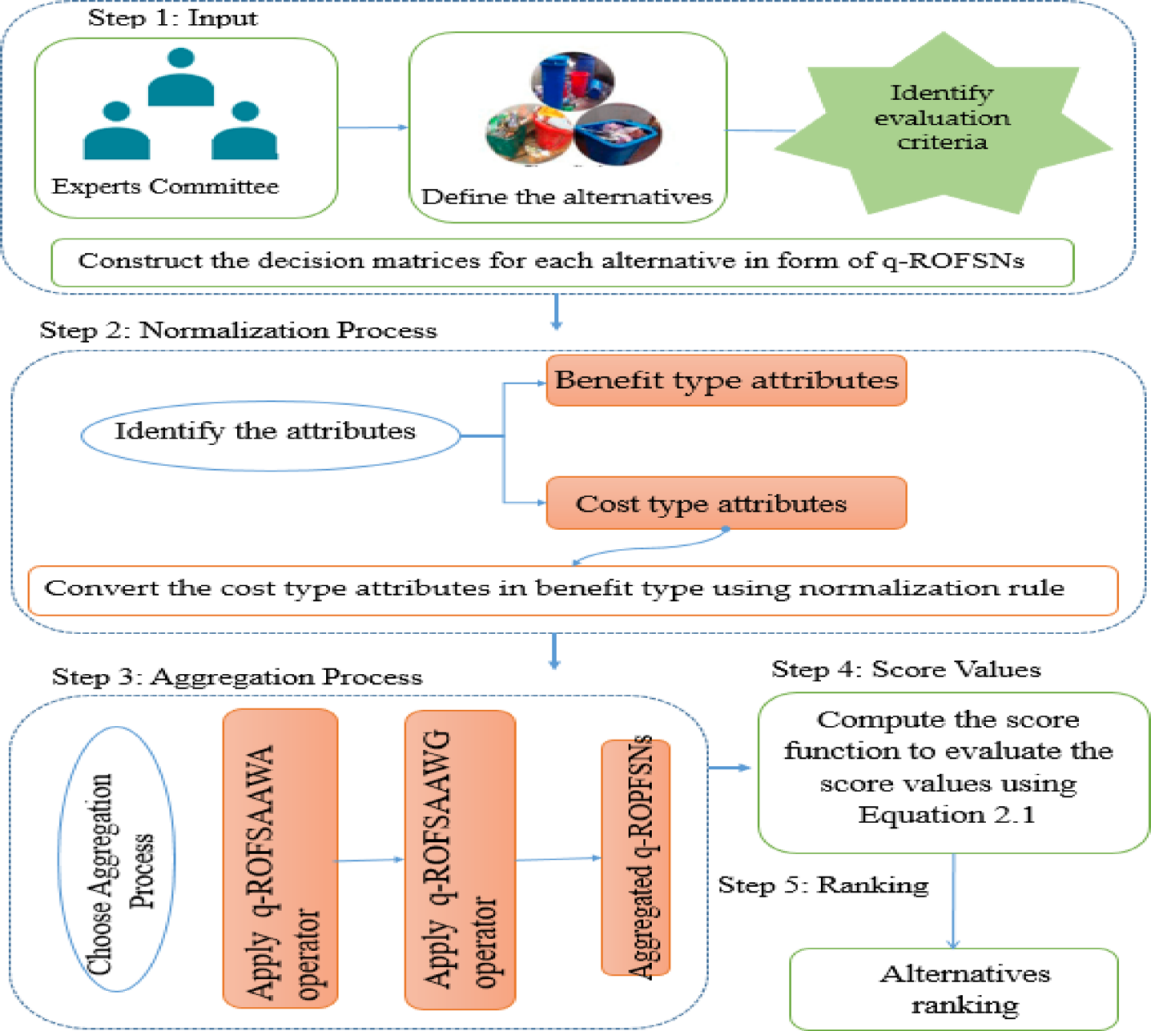
Flow chart of the proposed aczel–alsina-based MAGDM Model.

The visual description of the presented method is presented in Fig. 2.

## Selection of the food waste treatment technology using the proposed MAGDM model

This section poses a computing assessment focused on explaining the realistic practicality and import of the planned MAGDM technique in real-life decision-making.

### Food waste treatment technologies

Technologies for processing food waste play a vital role in alleviating the societal, financial, and social problems connected to food disposal. The exponential growth of worldwide food consumption and manufacturing has made food debris a critical environmental concern, exacerbating landfill collection, production of greenhouse gases, and the resulting depletion of scarce resources. Optimal techniques for treatment can reduce these challenges by minimizing the amount of rubbish, retrieving precious resources, and developing sustainable behaviors. The processing of wasted food generally boosts food availability by facilitating the equitable distribution of abundant or usable food to needy customers, minimizing hunger and relieving the total wasted weight. Moreover, these strategies can enhance a general understanding of the significance of reducing wasted food, resulting in enhanced conscientious consumption behaviors.

#### *Composting (*${{\mathcal{S}}}_{1}$*)*

Composting is an economical and sustainable technique for regulating food particles decomposing organic waste into nutritious composting that strengthens fertilization and layout. The strategy contains the degradation of food debris, plant contaminants, and different biodegradable substances by the microbes in oxygen-rich surroundings, facilitating the dissolution of more complicated biological substances into simple parts. The procedure often begins with collecting organic waste, which is then combined with carbon-rich supplies such as plants or bales to produce an appropriate carbon-to-nitrogen proportion, crucial for desirable microbial reproduction. Sufficient respiration is necessary to ensure adequate oxygen supply, hence expediting dissolution. The resultant composting is abundant in vital elements, including nitrogen, phosphorous, and magnesium, that enhance the development of plants and augment soil quality. Composting diminishes the quantity of trash directed to waste dumps, lowers methane emissions, and alleviates the impact of global warming. Moreover, it provides fiscal benefits by minimizing expenditures for disposal and diminishing the necessity for synthetic fertilizers. Also, composting promotes ecological farming, enhances awareness of the environment, and involves societies in more responsible waste-handling techniques. Community composting campaigns in towns and cities can function as teaching platforms, enabling citizens to influence their surroundings to decrease trash and conserve resources positively.

#### *Anaerobic digestion (*${{\mathcal{S}}}_{2}$*)*

Anaerobic digestion is a microbial method for managing food debris that evaporates in a devoid of oxygen in microbes that degrade organic compounds, including food debris, farming byproducts, and different recyclable materials. This technique generates biogas consisting of methane, which will be extracted and consumed as an alternate energy source for generating electricity and heating or as fuel for transportation. Anaerobic digestion develops renewable energy and a nutritious residue called waste products, which acts like a soil developer. Anaerobic digestion eliminates methane emissions, which could alternatively exacerbate environmental degradation, by transferring nutrients from dumpsters. This system efficiently manages food waste, encouraging waste elimination and energy utilization while stimulating ethical agricultural methods.

#### *Incineration (*${{\mathcal{S}}}_{3}$*)*

Incineration is a waste management strategy that signifies the thermal combustion of biological waste at scorching temperatures in a regulated configuration, transforming organic substances into debris, gases, and temperature. This procedure markedly decreases the volume of waste, rendering it an efficient approach to regulating substantial amounts of wasted food, particularly in regions with constrained landfill or composting capacity. Thermal energy produced throughout incineration can be utilized for energy production and converting garbage into a natural electricity source via waste-to-energy facilities. Nevertheless, although incineration decreases reliance on landfills and produces energy, it highlights apprehensions about environmental impact, especially releasing harmful gases and particle pollution. Conventional incineration plants are installed with sophisticated filtering techniques to reduce their ecological effects. The entire stability of the technique remains contentious because of its impact on the environment and the renewable energy characteristics of the procedure.

#### *Landfill (*${{\mathcal{S}}}_{4}$*)*

Landfilling is a commonly used technique for eliminating food debris related to the deposition of food residue with other organic remains in a specified location, usually a landfill, for prolonged decomposition. Eventually, the garbage encounters anaerobic decomposition, a technique devoid of oxygen, resulting in an output of methane, a potent, environmentally harmful gas. Even though landfilling is a facile and economical waste treatment process, it displays considerable environmental challenges, especially regarding methane production and harmful elements’ dissolution into neighbouring water in the ground. Contemporary dumpsters frequently incorporate gas extraction products to reduce methane production and avert contaminants. The technique is still more unstable than equivalents, such as composting or anaerobic digestion, that provide potential for resource extraction and diminish ecological effects.

**Fig. 3:**
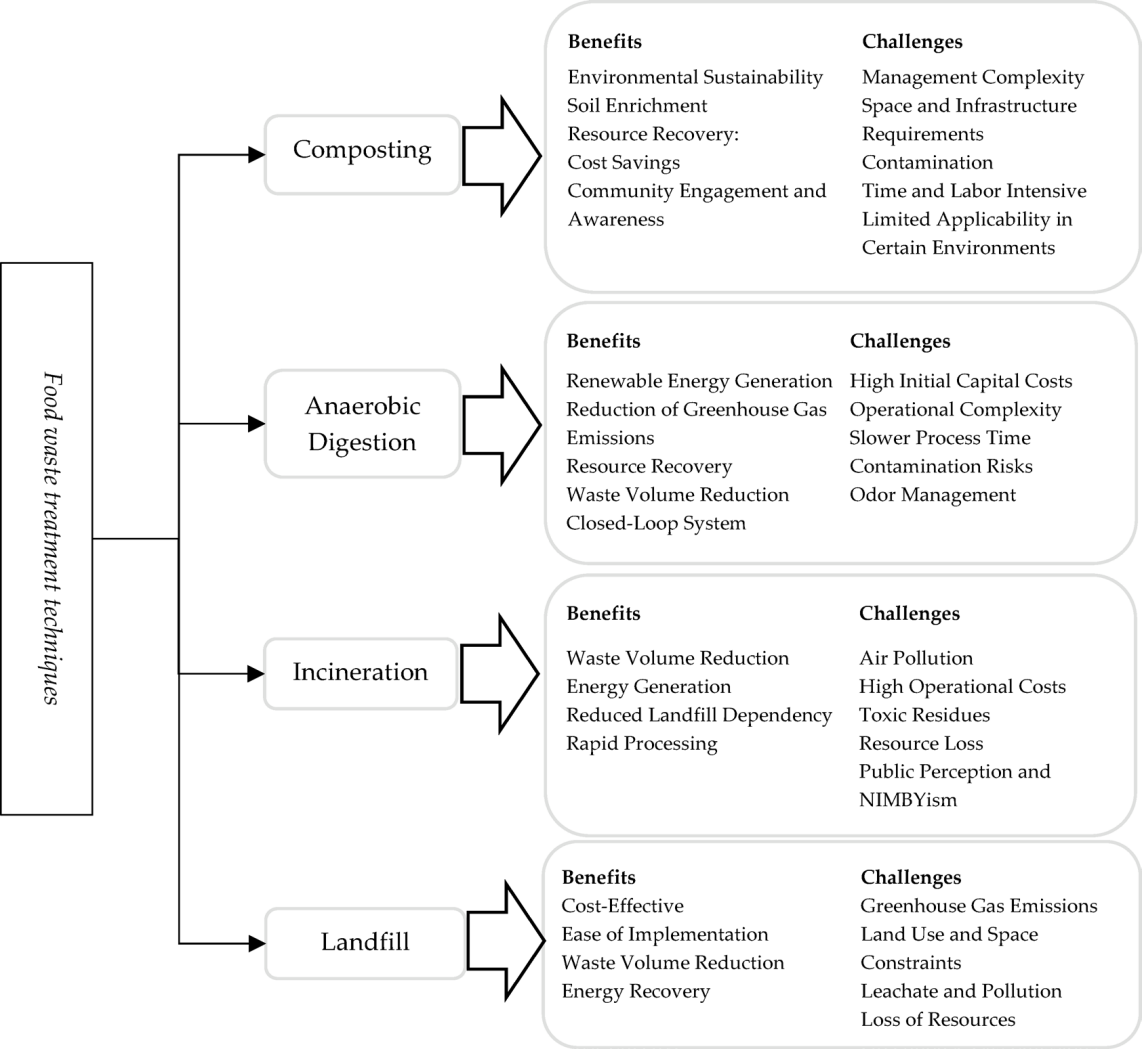
Food waste treatment techniques.

Figure 3, listed below, describes the potential benefits and drawbacks inherent in FWTT.

### Criteria description

#### Environmental impact

The conceivable natural complications of the extraction strategy, throughout released greenhouse gases, groundwater, and air degradation, and its potential to alleviate detrimental effects.

#### Resource recovery potential

The technique’s potential to regain significant resources, such as gas (biogas), composting, vital nutrients, or water, stimulates a circular economic system.

#### Waste reduction efficiency

This approach properly eliminates the quantity and mass of food debris, facilitating the administration of dumping prerequisites and landfill infrastructure.

#### Economic feasibility

The financing consumption, administrative costs, and hypothetical economic benefits or deposits corresponding to the execution and support of the treatment process.

#### Energy requirements and output

The electrical consumption of the operation is equated to the electricity restored or generated, which is apparent in anaerobic digestion or incineration technologies.

#### Scalability and flexibility

The technique is modular, determined by the amounts of food waste generated, and compatible with multiple waste formulations and geographical locations.

#### Regulatory compliance

Implementation of governmental, national, and global rules about the environment, waste disposal policies, and healthcare specifications.

#### Technical complexity and management

The algorithm’s operational complexities contain prerequisites for specialist expertise, administration expectations, and consistency of technological innovations.

#### Social acceptance and community impact

The completeness of community consensus to the recommended technique, integrating anxieties over fragrances, greenhouse gas emissions, and the closeness to domestic regions.

#### By-product management

The extent, excellence, and reliability of waste products produced (e.g., digestate, ash, leachate) and the practicability of their recycling, reusing, or controlled dumping.

A systematic study evaluation was carried out to rate the momentous state of FWTT and to find significant factors explored in prior research. In contrast, a panel of experts existed to investigate decision-making problems and discrepancies in this sector. Through an initial team of eleven specialists, five experts, each with at least ten years of professional experience and relevant certifications, were selected through a meticulous recruitment process to represent the final team of experts.

### Selection of food waste treatment technology

**Fig. 4:**
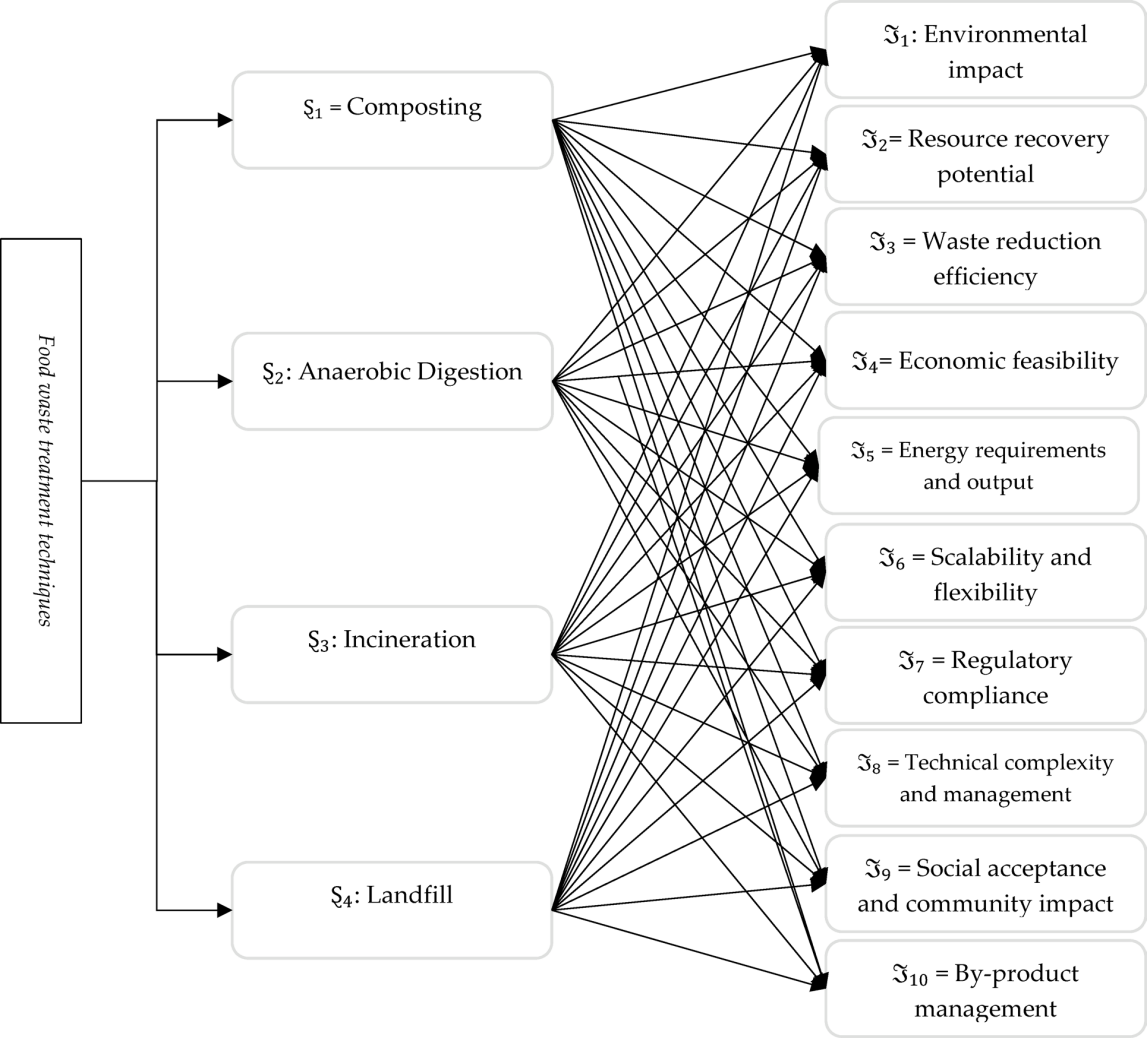
Selection criteria for food waste treatment technique.

The decision about the finest and most economical methodology for food waste processing is necessary to improve FWM policies. Subsection "Food waste treatment technologies" proposes a thorough investigation of that issue, concentrating on four treatment methods frequently utilized in FWM. The following section aims to determine a beneficial treatment approach and construct an organizational strategy to promote rational choices in selecting adequate procedures. Detailed specifications for each laid-out FWTT determined by this overview can be found as follows: ${\mathcal{S}}=$ {${{\mathcal{S}}}_{1}=$ Composting, ${{\mathcal{S}}}_{2}=$ Anaerobic Digestion, ${{\mathcal{S}}}_{3}=$ Incineration, ${{\mathcal{S}}}_{4}=$ Landfill}. Proper assessment factors are necessary for evaluating and selecting FWTT, supporting the experts in formulating accurate choices for the four suggested alternatives. In these circumstances, the following ten attributes will be addressed: $\mathfrak{I}=\{{\mathfrak{I}}_{1}=$ Environmental impact, ${\mathfrak{I}}_{2}=$ Resource recovery potential, ${\mathfrak{I}}_{3}=$ Waste reduction efficiency, ${\mathfrak{I}}_{4}=$ Economic feasibility, ${\mathfrak{I}}_{5}=$ Energy requirements and output, ${\mathfrak{I}}_{6}=$ Scalability and flexibility, ${\mathfrak{I}}_{7}=$ Regulatory compliance, ${\mathfrak{I}}_{8}=$ Technical complexity and management, ${\mathfrak{I}}_{9}=$ Social acceptance and community impact, ${\mathfrak{I}}_{10}=$ By-product management}. The weights of these considered factors are $\calligra{\rotatebox[origin=c]{22}{o}}=({0.15,0.07}, 0.09, 0.1, 0.12, 0.06, 0.05, 0.08, 0.12, 0.16),$ respectively, for the selection of FWTT. Let ${\mathcal{Z}}=\left\{{{\mathcal{Z}}}_{1}, {{\mathcal{Z}}}_{2}, {{\mathcal{Z}}}_{3}, {{\mathcal{Z}}}_{4}, {{\mathcal{Z}}}_{5}\right\}$ be a team of five experts whose weights are $\mathfrak{H}=(\text{0.22,0.19,0.18}, 0.20, 0.21)$. Figure 4 below expresses the logical characterization of parameters for deciding on FWTT.

The abovementioned approach encourages the establishment of the best and most economical alternative for food waste treatment by examining the factors listed above. The primary objective of contemporary FWTT is to establish economic sustainability that improves economic prosperity in the general framework of FWM. This study studies potential threats to human health and the surroundings, outlining the essential requirement to create outcomes that promote a stable society and sustainable development. Experts can ascertain the best optimal and economical methods for FWM by executing diligent investigations and examinations. Expert assessments were carried out using q-ROFSNs to evaluate every potential FWTT, as described in Table 1. The findings were integrated using the MAGDM technique, implementing the Aczel–Alsina AOs stated in Section "Aczel–Alsina weighted average aggregation operators for q-rung orthopair fuzzy soft sets" to find the most appropriate approach for FWM. The progressive methodology to determine the optimum solution via a proposed MAGDM methodology is presented as follows.

**Table 1:** Expert’s opinion for each food waste treatment technique in q-ROFSVs.

${{\mathcal{S}}}_{1}$	${\mathfrak{I}}_{1}$	${\mathfrak{I}}_{2}$	${\mathfrak{I}}_{3}$	${\mathfrak{I}}_{4}$	${\mathfrak{I}}_{5}$	${\mathfrak{I}}_{6}$	${\mathfrak{I}}_{7}$	${\mathfrak{I}}_{8}$	${\mathfrak{I}}_{9}$	${\mathfrak{I}}_{10}$
${{\mathcal{Z}}}_{1}$	$\left(\text{0.7,0.8}\right)$	$\left(\text{0.4,0.7}\right)$	$\left(\text{0.5,0.8}\right)$	$\left(0.7, 0.6\right)$	$\left(\text{0.4,0.9}\right)$	$\left(\text{0.8,0.7}\right)$	$\left(\text{0.5,0.8}\right)$	$\left(\text{0.5,0.7}\right)$	$\left(\text{0.7,0.6}\right)$	$\left(\text{0.5,0.7}\right)$
${{\mathcal{Z}}}_{2}$	$\left(\text{0.6,0.5}\right)$	$\left(\text{0.5,0.9}\right)$	$\left(\text{0.6,0.7}\right)$	$\left(\text{0.6,0.5}\right)$	$\left(\text{0.7,0.6}\right)$	$\left(\text{0.7,0.6}\right)$	$\left(\text{0.6,0.9}\right)$	$\left(\text{0.6,0.8}\right)$	$\left(\text{0.5,0.9}\right)$	$\left(\text{0.3,0.9}\right)$
${{\mathcal{Z}}}_{3}$	$\left(\text{0.4,0.7}\right)$	$\left(\text{0.6,0.7}\right)$	$\left(\text{0.5,0.9}\right)$	$\left(\text{0.8,0.5}\right)$	$\left(\text{0.6,0.8}\right)$	$\left(\text{0.6,0.8}\right)$	$\left(\text{0.7,0.8}\right)$	$\left(\text{0.6,0.5}\right)$	$\left(\text{0.6,0.8}\right)$	$\left(\text{0.6,0.8}\right)$
${{\mathcal{Z}}}_{4}$	$\left(\text{0.8,0.7}\right)$	$\left(\text{0.8,0.3}\right)$	$\left(\text{0.4,0.6}\right)$	$\left(\text{0.6,0.9}\right)$	$\left(\text{0.8,0.6}\right)$	$\left(\text{0.5,0.6}\right)$	$\left(\text{0.6,0.7}\right)$	$\left(\text{0.6,0.8}\right)$	$\left(\text{0.2,0.3}\right)$	$\left(\text{0.6,0.9}\right)$
${{\mathcal{Z}}}_{5}$	$\left(\text{0.6,0.8}\right)$	$\left(\text{0.7,0.8}\right)$	$\left(\text{0.6,0.7}\right)$	$\left(\text{0.3,0.8}\right)$	$\left(\text{0.7,0.8}\right)$	$\left(\text{0.7,0.8}\right)$	$\left(\text{0.8,0.3}\right)$	$\left(\text{0.7,0.8}\right)$	$\left(\text{0.7,0.8}\right)$	$\left(\text{0.5,0.8}\right)$
$${{\mathcal{S}}}_{2}$$										
${{\varvec{Z}}}_{1}$	$\left(0.7, 0.6\right)$	$\left(\text{0.4,0.9}\right)$	$\left(\text{0.8,0.7}\right)$	$\left(\text{0.5,0.8}\right)$	$\left(\text{0.7,0.6}\right)$	$\left(\text{0.7,0.8}\right)$	$\left(\text{0.4,0.7}\right)$	$\left(\text{0.5,0.8}\right)$	$\left(\text{0.5,0.7}\right)$	$\left(\text{0.5,0.7}\right)$
${{\mathcal{Z}}}_{2}$	$\left(\text{0.6,0.5}\right)$	$\left(\text{0.7,0.6}\right)$	$\left(\text{0.7,0.6}\right)$	$\left(\text{0.6,0.9}\right)$	$\left(\text{0.5,0.9}\right)$	$\left(\text{0.6,0.5}\right)$	$\left(\text{0.5,0.9}\right)$	$\left(\text{0.6,0.7}\right)$	$\left(\text{0.3,0.9}\right)$	$\left(\text{0.6,0.8}\right)$
${{\mathcal{Z}}}_{3}$	$\left(\text{0.8,0.5}\right)$	$\left(\text{0.6,0.8}\right)$	$\left(\text{0.6,0.8}\right)$	$\left(\text{0.7,0.8}\right)$	$\left(\text{0.6,0.8}\right)$	$\left(\text{0.4,0.7}\right)$	$\left(\text{0.6,0.7}\right)$	$\left(\text{0.5,0.9}\right)$	$\left(\text{0.6,0.8}\right)$	$\left(\text{0.6,0.5}\right)$
${{\mathcal{Z}}}_{4}$	$\left(\text{0.6,0.9}\right)$	$\left(\text{0.8,0.6}\right)$	$\left(\text{0.5,0.6}\right)$	$\left(\text{0.6,0.7}\right)$	$\left(\text{0.2,0.3}\right)$	$\left(\text{0.8,0.7}\right)$	$\left(\text{0.8,0.3}\right)$	$\left(\text{0.4,0.6}\right)$	$\left(\text{0.6,0.9}\right)$	$\left(\text{0.6,0.8}\right)$
${{\mathcal{Z}}}_{5}$	$\left(\text{0.3,0.8}\right)$	$\left(\text{0.7,0.8}\right)$	$\left(\text{0.7,0.8}\right)$	$\left(\text{0.8,0.3}\right)$	$\left(\text{0.7,0.8}\right)$	$\left(\text{0.6,0.8}\right)$	$\left(\text{0.7,0.8}\right)$	$\left(\text{0.6,0.7}\right)$	$\left(\text{0.5,0.8}\right)$	$\left(\text{0.7,0.8}\right)$
$${{\mathcal{S}}}_{3}$$										
${{\mathcal{Z}}}_{1}$	$\left(\text{0.6,0.9}\right)$	$\left(\text{0.8,0.6}\right)$	$\left(\text{0.5,0.6}\right)$	$\left(\text{0.6,0.7}\right)$	$\left(\text{0.2,0.3}\right)$	$\left(\text{0.8,0.7}\right)$	$\left(\text{0.8,0.3}\right)$	$\left(\text{0.4,0.6}\right)$	$\left(\text{0.6,0.9}\right)$	$\left(\text{0.6,0.8}\right)$
${{\mathcal{Z}}}_{2}$	$\left(\text{0.6,0.8}\right)$	$\left(\text{0.7,0.8}\right)$	$\left(\text{0.6,0.7}\right)$	$\left(\text{0.3,0.8}\right)$	$\left(\text{0.7,0.8}\right)$	$\left(\text{0.7,0.8}\right)$	$\left(\text{0.8,0.3}\right)$	$\left(\text{0.7,0.8}\right)$	$\left(\text{0.7,0.8}\right)$	$\left(\text{0.5,0.8}\right)$
${{\mathcal{Z}}}_{3}$	$\left(\text{0.7,0.8}\right)$	$\left(\text{0.4,0.7}\right)$	$\left(\text{0.5,0.8}\right)$	$\left(0.7, 0.6\right)$	$\left(\text{0.4,0.9}\right)$	$\left(\text{0.8,0.7}\right)$	$\left(\text{0.5,0.8}\right)$	$\left(\text{0.5,0.7}\right)$	$\left(\text{0.7,0.6}\right)$	$\left(\text{0.5,0.7}\right)$
${{\mathcal{Z}}}_{4}$	$\left(\text{0.6,0.5}\right)$	$\left(\text{0.7,0.6}\right)$	$\left(\text{0.7,0.6}\right)$	$\left(\text{0.6,0.9}\right)$	$\left(\text{0.5,0.9}\right)$	$\left(\text{0.6,0.5}\right)$	$\left(\text{0.5,0.9}\right)$	$\left(\text{0.6,0.7}\right)$	$\left(\text{0.3,0.9}\right)$	$\left(\text{0.6,0.8}\right)$
${{\mathcal{Z}}}_{5}$	$\left(\text{0.8,0.5}\right)$	$\left(\text{0.6,0.8}\right)$	$\left(\text{0.6,0.8}\right)$	$\left(\text{0.7,0.8}\right)$	$\left(\text{0.6,0.8}\right)$	$\left(\text{0.4,0.7}\right)$	$\left(\text{0.6,0.7}\right)$	$\left(\text{0.5,0.9}\right)$	$\left(\text{0.6,0.8}\right)$	$\left(\text{0.6,0.5}\right)$
$${{\mathcal{S}}}_{4}$$										
${{\mathcal{Z}}}_{1}$	$\left(\text{0.4,0.7}\right)$	$\left(\text{0.7,0.8}\right)$	$\left(\text{0.5,0.8}\right)$	$\left(\text{0.4,0.7}\right)$	$\left(\text{0.5,0.8}\right)$	$\left(\text{0.4,0.9}\right)$	$\left(\text{0.8,0.7}\right)$	$\left(\text{0.5,0.7}\right)$	$\left(\text{0.6,0.8}\right)$	$\left(\text{0.8,0.3}\right)$
${{\mathcal{Z}}}_{2}$	$\left(\text{0.5,0.9}\right)$	$\left(\text{0.6,0.5}\right)$	$\left(\text{0.6,0.7}\right)$	$\left(\text{0.5,0.9}\right)$	$\left(\text{0.6,0.7}\right)$	$\left(\text{0.7,0.6}\right)$	$\left(\text{0.7,0.6}\right)$	$\left(\text{0.3,0.9}\right)$	$\left(\text{0.1,0.7}\right)$	$\left(\text{0.8,0.3}\right)$
${{\mathcal{Z}}}_{3}$	$\left(\text{0.6,0.7}\right)$	$\left(\text{0.4,0.7}\right)$	$\left(\text{0.5,0.9}\right)$	$\left(\text{0.6,0.7}\right)$	$\left(\text{0.5,0.9}\right)$	$\left(\text{0.6,0.8}\right)$	$\left(\text{0.6,0.8}\right)$	$\left(\text{0.6,0.8}\right)$	$\left(\text{0.1,0.2}\right)$	$\left(\text{0.5,0.8}\right)$
${{\mathcal{Z}}}_{4}$	$\left(\text{0.8,0.3}\right)$	$\left(\text{0.8,0.7}\right)$	$\left(\text{0.4,0.6}\right)$	$\left(\text{0.8,0.3}\right)$	$\left(\text{0.4,0.6}\right)$	$\left(\text{0.8,0.6}\right)$	$\left(\text{0.5,0.6}\right)$	$\left(\text{0.6,0.9}\right)$	$\left(\text{0.3,0.8}\right)$	$\left(\text{0.5,0.9}\right)$
${{\mathcal{Z}}}_{5}$	$\left(\text{0.7,0.8}\right)$	$\left(\text{0.6,0.8}\right)$	$\left(\text{0.6,0.7}\right)$	$\left(\text{0.7,0.8}\right)$	$\left(\text{0.6,0.7}\right)$	$\left(\text{0.7,0.8}\right)$	$\left(\text{0.7,0.8}\right)$	$\left(\text{0.5,0.8}\right)$	$\left(\text{0.4,0.9}\right)$	$\left(\text{0.6,0.7}\right)$

#### Selection of food waste treatment technique using q-ROFSAAWA operator

Step 1: An advisory board of experts engaged in a detailed analysis of every prospective FWTT and reported their opinions using a profound exploration. The professionals’ opinions of each alternate, formulated by employing q-ROFSNs, are highlighted in Table 1.

Step 2: In relation to ${\mathfrak{I}}_{4}$ (Economic Feasibility) as a cost-related factor, the normalization technique will be implemented according to the recommendation in Eq. 4.1. This regulation supports the effective optimization of the factor and its modification into a beneficial shape for impartial screening. The modified normalized outcomes are listed in Table 2.

**Table 2:** Normalized decision matrices.

${{\mathcal{S}}}_{1}$	${\mathfrak{I}}_{1}$	${\mathfrak{I}}_{2}$	${\mathfrak{I}}_{3}$	${\mathfrak{I}}_{4}$	${\mathfrak{I}}_{5}$	${\mathfrak{I}}_{6}$	${\mathfrak{I}}_{7}$	${\mathfrak{I}}_{8}$	${\mathfrak{I}}_{9}$	${\mathfrak{I}}_{10}$
${{\mathcal{Z}}}_{1}$	$\left(\text{0.7,0.8}\right)$	$\left(\text{0.4,0.7}\right)$	$\left(\text{0.5,0.8}\right)$	$\left(0.6, 0.7\right)$	$\left(\text{0.4,0.9}\right)$	$\left(\text{0.8,0.7}\right)$	$\left(\text{0.5,0.8}\right)$	$\left(\text{0.5,0.7}\right)$	$\left(\text{0.7,0.6}\right)$	$\left(\text{0.5,0.7}\right)$
${{\mathcal{Z}}}_{2}$	$\left(\text{0.6,0.5}\right)$	$\left(\text{0.5,0.9}\right)$	$\left(\text{0.6,0.7}\right)$	$\left(0.5, 0.6\right)$	$\left(\text{0.7,0.6}\right)$	$\left(\text{0.7,0.6}\right)$	$\left(\text{0.6,0.9}\right)$	$\left(\text{0.6,0.8}\right)$	$\left(\text{0.5,0.9}\right)$	$\left(\text{0.3,0.9}\right)$
${{\mathcal{Z}}}_{3}$	$\left(\text{0.4,0.7}\right)$	$\left(\text{0.6,0.7}\right)$	$\left(\text{0.5,0.9}\right)$	$\left(0.5, 0.8\right)$	$\left(\text{0.6,0.8}\right)$	$\left(\text{0.6,0.8}\right)$	$\left(\text{0.7,0.8}\right)$	$\left(\text{0.6,0.5}\right)$	$\left(\text{0.6,0.8}\right)$	$\left(\text{0.6,0.8}\right)$
${{\mathcal{Z}}}_{4}$	$\left(\text{0.8,0.7}\right)$	$\left(\text{0.8,0.3}\right)$	$\left(\text{0.4,0.6}\right)$	$\left(0.9, 0.6\right)$	$\left(\text{0.8,0.6}\right)$	$\left(\text{0.5,0.6}\right)$	$\left(\text{0.6,0.7}\right)$	$\left(\text{0.6,0.8}\right)$	$\left(\text{0.2,0.3}\right)$	$\left(\text{0.6,0.9}\right)$
${{\mathcal{Z}}}_{5}$	$\left(\text{0.6,0.8}\right)$	$\left(\text{0.7,0.8}\right)$	$\left(\text{0.6,0.7}\right)$	$\left(0.8, 0.3\right)$	$\left(\text{0.7,0.8}\right)$	$\left(\text{0.7,0.8}\right)$	$\left(\text{0.8,0.3}\right)$	$\left(\text{0.7,0.8}\right)$	$\left(\text{0.7,0.8}\right)$	$\left(\text{0.5,0.8}\right)$
$${{\mathcal{S}}}_{2}$$										
${{\mathcal{Z}}}_{1}$	$\left(0.7, 0.6\right)$	$\left(\text{0.4,0.9}\right)$	$\left(\text{0.8,0.7}\right)$	$\left(\text{0.8,0.5}\right)$	$\left(\text{0.7,0.6}\right)$	$\left(\text{0.7,0.8}\right)$	$\left(\text{0.4,0.7}\right)$	$\left(\text{0.5,0.8}\right)$	$\left(\text{0.5,0.7}\right)$	$\left(\text{0.5,0.7}\right)$
${{\mathcal{Z}}}_{2}$	$\left(\text{0.6,0.5}\right)$	$\left(\text{0.7,0.6}\right)$	$\left(\text{0.7,0.6}\right)$	$\left(\text{0.9,0.6}\right)$	$\left(\text{0.5,0.9}\right)$	$\left(\text{0.6,0.5}\right)$	$\left(\text{0.5,0.9}\right)$	$\left(\text{0.6,0.7}\right)$	$\left(\text{0.3,0.9}\right)$	$\left(\text{0.6,0.8}\right)$
${{\mathcal{Z}}}_{3}$	$\left(\text{0.8,0.5}\right)$	$\left(\text{0.6,0.8}\right)$	$\left(\text{0.6,0.8}\right)$	$\left(\text{0.8,0.7}\right)$	$\left(\text{0.6,0.8}\right)$	$\left(\text{0.4,0.7}\right)$	$\left(\text{0.6,0.7}\right)$	$\left(\text{0.5,0.9}\right)$	$\left(\text{0.6,0.8}\right)$	$\left(\text{0.6,0.5}\right)$
${{\mathcal{Z}}}_{4}$	$\left(\text{0.6,0.9}\right)$	$\left(\text{0.8,0.6}\right)$	$\left(\text{0.5,0.6}\right)$	$\left(\text{0.7,0.6}\right)$	$\left(\text{0.2,0.3}\right)$	$\left(\text{0.8,0.7}\right)$	$\left(\text{0.8,0.3}\right)$	$\left(\text{0.4,0.6}\right)$	$\left(\text{0.6,0.9}\right)$	$\left(\text{0.6,0.8}\right)$
${{\mathcal{Z}}}_{5}$	$\left(\text{0.3,0.8}\right)$	$\left(\text{0.7,0.8}\right)$	$\left(\text{0.7,0.8}\right)$	$\left(\text{0.3,0.8}\right)$	$\left(\text{0.7,0.8}\right)$	$\left(\text{0.6,0.8}\right)$	$\left(\text{0.7,0.8}\right)$	$\left(\text{0.6,0.7}\right)$	$\left(\text{0.5,0.8}\right)$	$\left(\text{0.7,0.8}\right)$
$${{\mathcal{S}}}_{3}$$										
${{\mathcal{Z}}}_{1}$	$\left(\text{0.6,0.9}\right)$	$\left(\text{0.8,0.6}\right)$	$\left(\text{0.5,0.6}\right)$	$\left(\text{0.7,0.6}\right)$	$\left(\text{0.2,0.3}\right)$	$\left(\text{0.8,0.7}\right)$	$\left(\text{0.8,0.3}\right)$	$\left(\text{0.4,0.6}\right)$	$\left(\text{0.6,0.9}\right)$	$\left(\text{0.6,0.8}\right)$
${{\mathcal{Z}}}_{2}$	$\left(\text{0.6,0.8}\right)$	$\left(\text{0.7,0.8}\right)$	$\left(\text{0.6,0.7}\right)$	$\left(\text{0.8,0.3}\right)$	$\left(\text{0.7,0.8}\right)$	$\left(\text{0.7,0.8}\right)$	$\left(\text{0.8,0.3}\right)$	$\left(\text{0.7,0.8}\right)$	$\left(\text{0.7,0.8}\right)$	$\left(\text{0.5,0.8}\right)$
${{\mathcal{Z}}}_{3}$	$\left(\text{0.7,0.8}\right)$	$\left(\text{0.4,0.7}\right)$	$\left(\text{0.5,0.8}\right)$	$\left(0.6, 0.7\right)$	$\left(\text{0.4,0.9}\right)$	$\left(\text{0.8,0.7}\right)$	$\left(\text{0.5,0.8}\right)$	$\left(\text{0.5,0.7}\right)$	$\left(\text{0.7,0.6}\right)$	$\left(\text{0.5,0.7}\right)$
${{\mathcal{Z}}}_{4}$	$\left(\text{0.6,0.5}\right)$	$\left(\text{0.7,0.6}\right)$	$\left(\text{0.7,0.6}\right)$	$\left(\text{0.9,0.6}\right)$	$\left(\text{0.5,0.9}\right)$	$\left(\text{0.6,0.5}\right)$	$\left(\text{0.5,0.9}\right)$	$\left(\text{0.6,0.7}\right)$	$\left(\text{0.3,0.9}\right)$	$\left(\text{0.6,0.8}\right)$
${{\mathcal{Z}}}_{5}$	$\left(\text{0.8,0.5}\right)$	$\left(\text{0.6,0.8}\right)$	$\left(\text{0.6,0.8}\right)$	$\left(\text{0.8,0.7}\right)$	$\left(\text{0.6,0.8}\right)$	$\left(\text{0.4,0.7}\right)$	$\left(\text{0.6,0.7}\right)$	$\left(\text{0.5,0.9}\right)$	$\left(\text{0.6,0.8}\right)$	$\left(\text{0.6,0.5}\right)$
$${{\mathcal{S}}}_{4}$$										
${{\mathcal{Z}}}_{1}$	$\left(\text{0.4,0.7}\right)$	$\left(\text{0.7,0.8}\right)$	$\left(\text{0.5,0.8}\right)$	$\left(\text{0.7,0.4}\right)$	$\left(\text{0.5,0.8}\right)$	$\left(\text{0.4,0.9}\right)$	$\left(\text{0.8,0.7}\right)$	$\left(\text{0.5,0.7}\right)$	$\left(\text{0.6,0.8}\right)$	$\left(\text{0.8,0.3}\right)$
${{\mathcal{Z}}}_{2}$	$\left(\text{0.5,0.9}\right)$	$\left(\text{0.6,0.5}\right)$	$\left(\text{0.6,0.7}\right)$	$\left(\text{0.9,0.5}\right)$	$\left(\text{0.6,0.7}\right)$	$\left(\text{0.7,0.6}\right)$	$\left(\text{0.7,0.6}\right)$	$\left(\text{0.3,0.9}\right)$	$\left(\text{0.1,0.7}\right)$	$\left(\text{0.8,0.3}\right)$
${{\mathcal{Z}}}_{3}$	$\left(\text{0.6,0.7}\right)$	$\left(\text{0.4,0.7}\right)$	$\left(\text{0.5,0.9}\right)$	$\left(\text{0.7,0.6}\right)$	$\left(\text{0.5,0.9}\right)$	$\left(\text{0.6,0.8}\right)$	$\left(\text{0.6,0.8}\right)$	$\left(\text{0.6,0.8}\right)$	$\left(\text{0.1,0.2}\right)$	$\left(\text{0.5,0.8}\right)$
${{\mathcal{Z}}}_{4}$	$\left(\text{0.8,0.3}\right)$	$\left(\text{0.8,0.7}\right)$	$\left(\text{0.4,0.6}\right)$	$\left(\text{0.3,0.8}\right)$	$\left(\text{0.4,0.6}\right)$	$\left(\text{0.8,0.6}\right)$	$\left(\text{0.5,0.6}\right)$	$\left(\text{0.6,0.9}\right)$	$\left(\text{0.3,0.8}\right)$	$\left(\text{0.5,0.9}\right)$
${{\mathcal{Z}}}_{5}$	$\left(\text{0.7,0.8}\right)$	$\left(\text{0.6,0.8}\right)$	$\left(\text{0.6,0.7}\right)$	$\left(\text{0.8,0.7}\right)$	$\left(\text{0.6,0.7}\right)$	$\left(\text{0.7,0.8}\right)$	$\left(\text{0.7,0.8}\right)$	$\left(\text{0.5,0.8}\right)$	$\left(\text{0.4,0.9}\right)$	$\left(\text{0.6,0.7}\right)$

Step 3: The accumulated preference scores for all alternatives are derived by implementing Eq. 3.1. The subsequent cumulative results are listed in the following way: ${\upzeta }_{1}=\left(0.54462, 0.526374\right)$, ${\upzeta }_{2}=\left(0.505620, 0.475484\right)$, ${\upzeta }_{3}=\left(0.558035, 0.504721\right)$, and ${\upzeta }_{4}=\left(0.449529, 0.494924\right)$.

Step 4: We will apply the score function stated in Eq. 2.1 to identify the most appropriate food waste treatment technique. The obtained score values are given as $\Psi \left({\upzeta }_{1}\right)=0.018252$, $\Psi \left({\upzeta }_{2}\right)=0.025915$, $\Psi \left({\upzeta }_{3}\right)=0.053081$, and $\Psi \left({\upzeta }_{4}\right)=-0.036378$.

Step 5: An alternative with the largest determined score constitutes the most efficient FWTT. Meanwhile, the alternate with the lowest value is perceived as poor. Alternatives are organized in ascending order considering their score values, with greater scores demonstrating better implications. For instance, the evaluation can be described as: $\Psi \left({\upzeta }_{3}\right)>\Psi \left({\upzeta }_{2}\right)>\Psi \left({\upzeta }_{1}\right)>\Psi \left({\upzeta }_{4}\right)$.

Step 6: The alternatives are listed in ascending order, determined by their generated score values. This classification implies the relative merits of each FWTT, with a higher score demonstrating the best choice. This shows that the ${{\mathcal{S}}}_{3}$ (Incineration) be the most effective FWTT in food waste management. The logical assessment of possible outcomes is as follows: ${{\mathcal{S}}}_{3}>{{\mathcal{S}}}_{2}>{{\mathcal{S}}}_{1}>{{\mathcal{S}}}_{4}$.

#### Selection of food waste treatment technique using q-ROFSAAWG operator

Steps 1 and 2 are the same as the subsection "Selection of food waste treatment technique using q-ROFSAAWA operator".

Step 3: The accumulated preference scores for all alternatives are derived by implementing Eq. 3.2. The subsequent cumulative results are listed in the following way: ${\upzeta }_{1}=\left(0.599605, 0.877174\right)$, ${\upzeta }_{2}=\left(0.590356, 0.880350\right)$, ${\upzeta }_{3}=\left(0.592273, 0.858192\right)$, and ${\upzeta }_{4}=\left(0.594716, 0.865132\right)$.

Step 4: We will apply the score function stated in Eq. 2.1 to identify the most appropriate food waste treatment technique. The obtained score values are given as $\Psi \left({\upzeta }_{1}\right)=-0.578591$, $\Psi \left({\upzeta }_{2}\right)=-0.613640$, $\Psi \left({\upzeta }_{3}\right)=-0.555141$, and $\Psi \left({\upzeta }_{4}\right)=-0.568486$.

Step 5: An alternative with the largest determined score constitutes the most efficient FWTT. Meanwhile, the alternate with the lowest value is perceived as poor. Alternatives are organized in ascending order considering their score values, with greater scores demonstrating better implications. For instance, the evaluation can be described as: $\Psi \left({\upzeta }_{3}\right)>\Psi \left({\upzeta }_{4}\right)>\Psi \left({\upzeta }_{1}\right)>\Psi \left({\upzeta }_{2}\right)$.

Step 6: The alternatives are listed in ascending order, determined by their generated score values. This classification implies the relative merits of each FWTT, with a higher score demonstrating the best choice. This shows that the ${{\mathcal{S}}}_{3}$ (Incineration) be the most effective FWTT in food waste management. The logical assessment of possible outcomes is as follows: ${{\mathcal{S}}}_{3}>{{\mathcal{S}}}_{4}>{{\mathcal{S}}}_{1}>{{\mathcal{S}}}_{2}$.

Table 3 below demonstrates the alternates’ score values and ranking extracted using the q-ROFSAAWA and q-ROFSAAWG operators.

**Table 3:** Ranking order of the considered alternatives using the proposed Aczel Alsina AOs.

Operator	$\Psi \left({\upzeta }_{1}\right)$	$\Psi \left({\upzeta }_{2}\right)$	$\Psi \left({\upzeta }_{3}\right)$	$\Psi \left({\upzeta }_{4}\right)$	Alternatives ranking
q-ROFSAAWA operator	0.018252	0.025915	0.053081	− 0.036378	${{\mathcal{S}}}_{3}>{{\mathcal{S}}}_{2}>{{\mathcal{S}}}_{1}>{{\mathcal{S}}}_{4}$
q-ROFSAAWG operator	− 0.578591	− 0.613440	− 0.555141	− 0.568486	${{\mathcal{S}}}_{3}>{{\mathcal{S}}}_{4}>{{\mathcal{S}}}_{1}>{{\mathcal{S}}}_{2}$

**Fig. 5:**
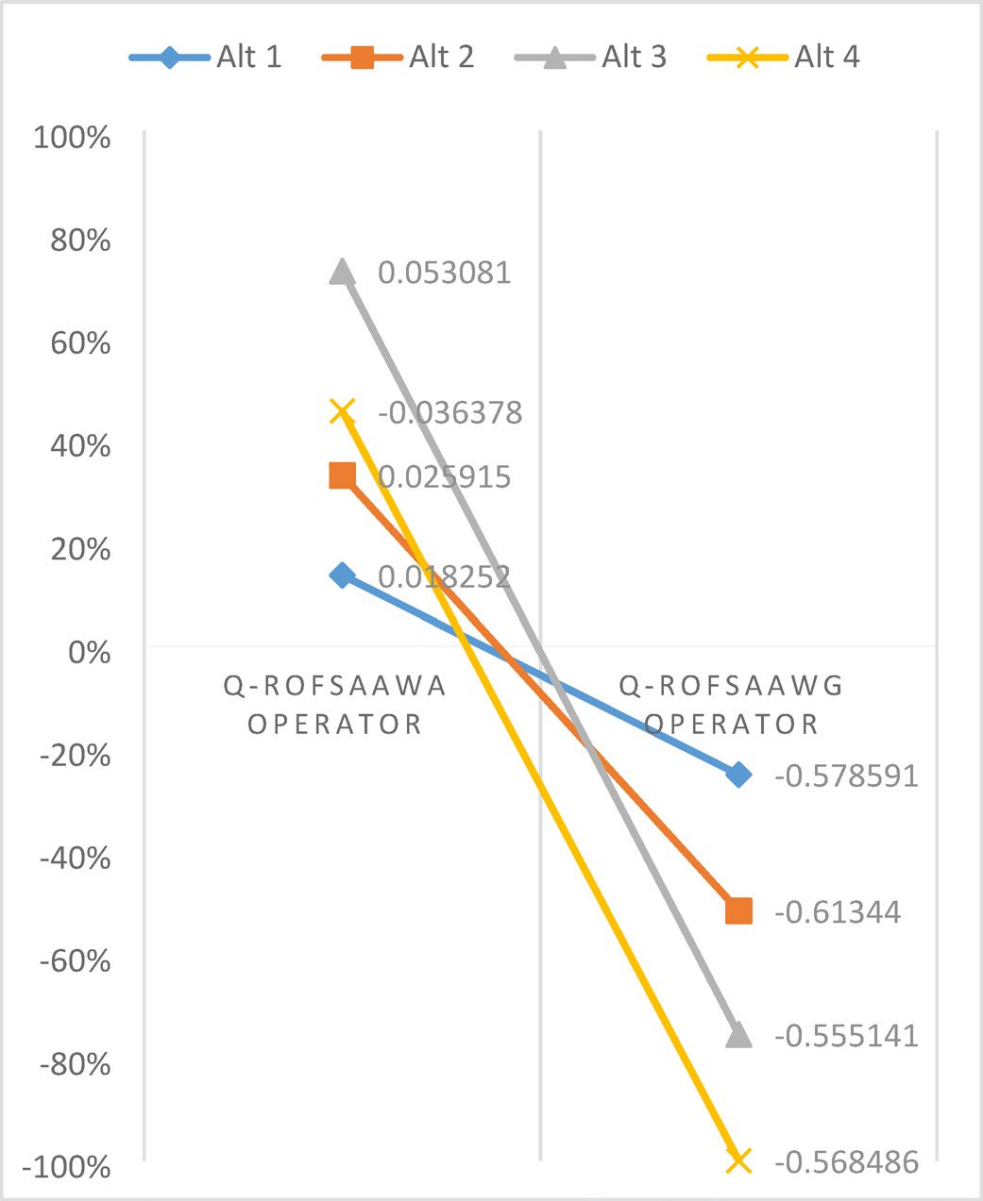
Alternatives ranking using proposed operators.

Figure 5 below highlights a visualization of the constructed q-ROFSAAWA and q-ROFSAAWG operators.

Evaluation of a compatible MAGDM system is imperative for an organization’s growth. Our exciting structure delicately scrutinizes every important aspect of that problem, stimulating the experts to develop more balanced and logical feedback. This method stated in this investigation will be pragmatic and financially notable. The q-ROFSAAWA and q-ROFSAAWG operators increase an exciting MAGDM mechanism that incorporates both operators’ beneficial characteristics and capacities. This technique presents significant benefits compared to traditional methodologies in managing those problems associated with MAGDM. It boosts mobility by including expulsion, solidity, and modification to accommodate different situations.

## Sensitivity and comparative analysis

To present the developed strategy’s integrity and potency, we evaluate the prescribed AOs’ sensitivity. Moreover, we execute comparisons from three different perspectives, such as the Aczel–Alsina AOs in different frameworks, AOs with identical structures, and different decision-making models in the same structure.

### Sensitivity analysis

**Fig. 6:**
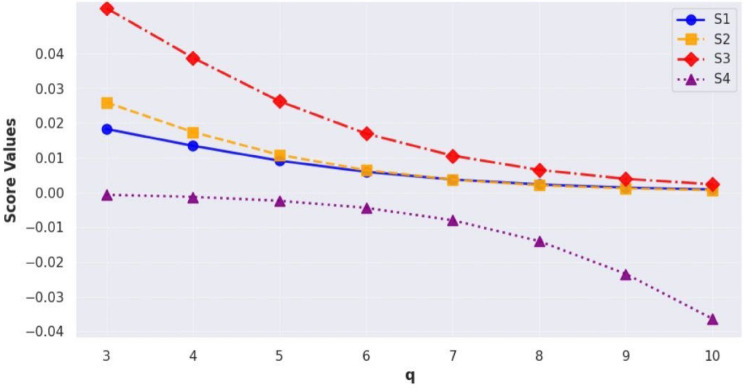
Sensitivity analysis using q-ROFSAAWA operator.

The influence of manipulating the rung parameter $q$ in the q-ROFSAAWA operator on the ranking findings is shown in Table 4. Figure 6 indicates the animated illustrations of Table 4, presenting different configurations of the $q$ parameter for both operators. Table 4, as well as Fig. 6, confirm that although the order of preference fluctuates a little with varying values of $q$, the optimum option, ${{\mathcal{S}}}_{3}$ is stable for the q-ROFSAAWA operator.

**Table 4:** Effect on the alternatives ranking by the variation of $q$ using q-ROFSAAWA operator.

Value of $q$	$\Psi \left({\upzeta }_{1}\right)$	$\Psi \left({\upzeta }_{2}\right)$	$\Psi \left({\upzeta }_{3}\right)$	$\Psi \left({\upzeta }_{4}\right)$	Ranking
$q=3$	0.018252	0.025915	0.053081	− 0.000681	${{\mathcal{S}}}_{3}>{{\mathcal{S}}}_{2}>{{\mathcal{S}}}_{1}>{{\mathcal{S}}}_{4}$
$q=4$	0.013428	0.017389	0.038789	− 0.001290	${{\mathcal{S}}}_{3}>{{\mathcal{S}}}_{2}>{{\mathcal{S}}}_{1}>{\mathcal{S}}_{4}$
$q=5$	0.009132	0.010802	0.026236	− 0.002413	${{\mathcal{S}}}_{3}>{{\mathcal{S}}}_{2}>{{\mathcal{S}}}_{1}>{\mathcal{S}}_{4}$
$q=6$	0.005918	0.006404	0.016923	− 0.004446	${{\mathcal{S}}}_{3}>{{\mathcal{S}}}_{2}>{{\mathcal{S}}}_{1}>{\mathcal{S}}_{4}$
$q=7$	0.003715	0.003682	0.010581	− 0.008020	${{\mathcal{S}}}_{3}>{{\mathcal{S}}}_{1}>{{\mathcal{S}}}_{2}>{\mathcal{S}}_{4}$
$q=8$	0.002280	0.002071	0.006473	− 0.014037	${{\mathcal{S}}}_{3}>{{\mathcal{S}}}_{1}>{{\mathcal{S}}}_{2}>{\mathcal{S}}_{4}$
$q=9$	0.001376	0.001146	0.003897	− 0.023473	${{\mathcal{S}}}_{3}>{{\mathcal{S}}}_{1}>{{\mathcal{S}}}_{2}>{\mathcal{S}}_{4}$
$q=10$	0.000820	0.000626	0.002318	− 0.036378	${{\mathcal{S}}}_{3}>{{\mathcal{S}}}_{1}>{{\mathcal{S}}}_{2}>{\mathcal{S}}_{4}$

Also, Fig. 6 and Table 4 clarify an ongoing fall in numerical scores as the index of $q$ goes in both operators.

**Fig. 7:**
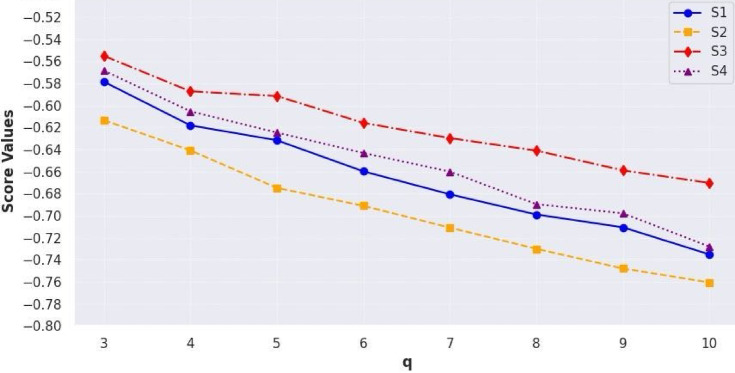
Sensitivity analysis using q-ROFSAAWG operator.

The influence of manipulating the rung parameter $q$ in the q-ROFSAAWG operator on the ranking findings is shown in Table 5. Figure 7 indicates the animated illustrations of this table, presenting different configurations of the $q$ parameter for the q-ROFSAAWG operator. Table 5, as well as Fig. 7, confirm that although the order of preference fluctuates a little with varying values of $q$, the optimum option, ${{\mathcal{S}}}_{3}$ is stable for the q-ROFSAAWG operator.

**Table 5:** Effect on the alternatives ranking by the variation of $q$ using q-ROFSAAWG operator.

Value of $q$	$\Psi \left({\upzeta }_{1}\right)$	$\Psi \left({\upzeta }_{2}\right)$	$\Psi \left({\upzeta }_{3}\right)$	$\Psi \left({\upzeta }_{4}\right)$	ranking
$q=3$	− 0.578591	− 0.613440	− 0.555141	− 0.568486	${{\mathcal{S}}}_{3}>{{\mathcal{S}}}_{4}>{{\mathcal{S}}}_{1}>{\mathcal{S}}_{2}$
$q=4$	− 0.618146	− 0.640842	− 0.587291	− 0.605419	${{\mathcal{S}}}_{3}>{{\mathcal{S}}}_{4}>{{\mathcal{S}}}_{1}>{\mathcal{S}}_{2}$
$q=5$	− 0.631672	− 0.674851	− 0.591582	− 0.624653	${{\mathcal{S}}}_{3}>{{\mathcal{S}}}_{4}>{{\mathcal{S}}}_{1}>{\mathcal{S}}_{2}$
$q=6$	− 0.659836	− 0.691096	− 0.615927	− 0.643190	${{\mathcal{S}}}_{3}>{{\mathcal{S}}}_{4}>{{\mathcal{S}}}_{1}>{\mathcal{S}}_{2}$
$q=7$	− 0.680659	− 0.710916	− 0.629707	− 0.660085	${{\mathcal{S}}}_{3}>{{\mathcal{S}}}_{4}>{{\mathcal{S}}}_{1}>{\mathcal{S}}_{2}$
$q=8$	− 0.698991	− 0.730085	− 0.641073	− 0.689583	${{\mathcal{S}}}_{3}>{{\mathcal{S}}}_{4}>{{\mathcal{S}}}_{1}>{\mathcal{S}}_{2}$
$q=9$	− 0.710586	− 0.747951	− 0.658959	− 0.697854	${{\mathcal{S}}}_{3}>{{\mathcal{S}}}_{4}>{{\mathcal{S}}}_{1}>{\mathcal{S}}_{2}$
$q=10$	− 0.735169	− 0.760563	− 0.670384	− 0.728049	${{\mathcal{S}}}_{3}>{{\mathcal{S}}}_{4}>{{\mathcal{S}}}_{1}>{\mathcal{S}}_{2}$

Also, Fig. 7 and Table 5 clarify an ongoing fall in numerical scores as the index of $q$ goes in both operators.

### Comparison with Aczel–Alsina aggregation operators in different structures

The validity of the aggregation-based MAGDM method is evidenced by its exclusive advantages, which are distinguished from previous research. The comparison of facts proved that the outcomes drawn from this method resembled those extracted from standard techniques. This methodology’s core vitality is its tendency to apply more detail describing restrictions in various categories, thus substantially alleviating data obscurity. Therefore, it delivers an improved and precise representation of multiple factors, rendering it an invaluable instrument for acquiring necessary data to facilitate decisions.

The MAGDM methodology, confined to Aczel–Alsina, properly covers the fundamental problems that Aczel–Alsina concepts face, particularly with managing multiple variations. Its utility is determined by its potential to locate the optimum FWTT in FWM. Table 6 represents the ability of the Aczel–Alsina operators performed in the q-ROFSS structure to exceed other strategies in recognizing the most effective decision from all possibilities.

**Table 6:** Comparison of aczel–alsina AOs in different structures.

Structure	Operator	Alternatives score values				Ranking
IFS^29^	Aczel–alsina	n/a				n/a
IHFS^31^	Aczel–alsina	n/a				n/a
CIFS^32^	Aczel–alsina	n/a				n/a
PyFS^35^	Aczel–alsina	n/a				n/a
CPyFS^38^	Aczel–alsina	n/a				n/a
q-ROFS^39^	Aczel–alsina	n/a				n/a
q-ROFSS	q-ROFSAAWA operator	0.018252	0.025915	0.053081	− 0.036378	${{\mathcal{S}}}_{3}>{{\mathcal{S}}}_{2}>{{\mathcal{S}}}_{1}>{\mathcal{S}}_{4}$
q-ROFSS	q-ROFSAAWG operator	− 0.578591	− 0.613440	− 0.555141	− 0.568486	${{\mathcal{S}}}_{3}>{{\mathcal{S}}}_{4}>{{\mathcal{S}}}_{1}>{\mathcal{S}}_{2}$

### Comparison of the proposed operators with different aggregation operators in the same structure

**Fig. 8:**
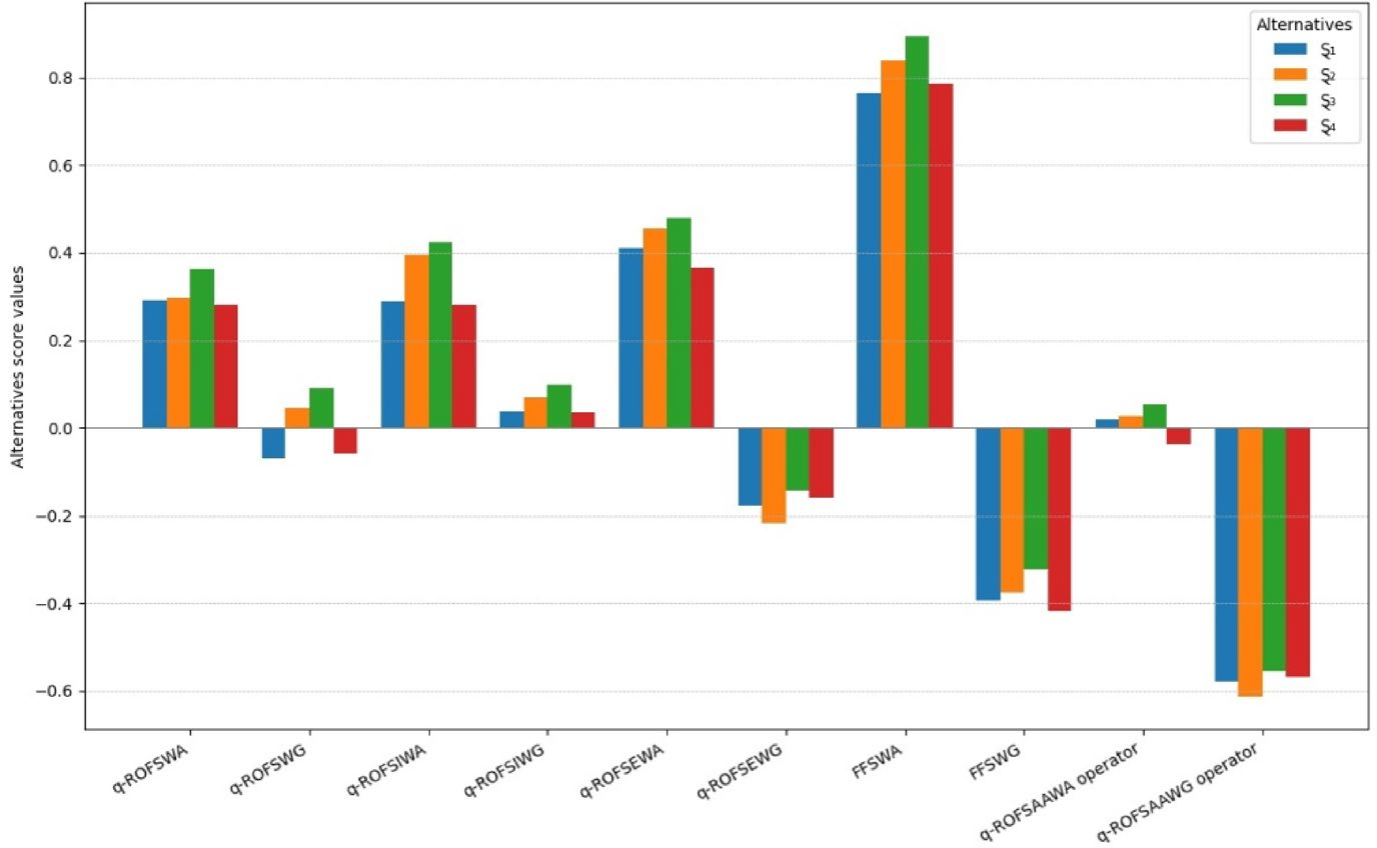
Comparison with different aggregation operators in the same structure.

An evaluation is executed from an assessment of AOs to demonstrate the potential benefits of the recommended system. This strategy is compared by exploiting different operators, such as q-ROFSWA^29^, q-ROFSWG^44^, q-ROFSIWA^46^, q-ROFSIWG^46^, q-ROFSEWA^47^, q-ROFSEWG^48^, Fermatean fuzzy soft weighted average (FFSWA)^51^ and Fermatean fuzzy soft weighted geometric (FFSWG)^51^ operators. The ranking outcomes derived from different operators are provided in Table 7 and Fig. 8. The specifications for assigning FWTT fluctuate and are determined by the aggregation approaches implemented to collect and assess the facts, as represented in Table 7. Despite these disparities, the ultimate assessment continues to be consistent. As an example, ${\mathcal{S}}$₃ is frequently viewed as one of the best and most viable FWTT alternatives.

**Table 7:** Comparison with different AOs.

Operator	Alternatives score values				Ranking
q-ROFSWA^29^	0.290185	0.296543	0.362914	0.281573	${{\mathcal{S}}}_{3}>{{\mathcal{S}}}_{2}>{{\mathcal{S}}}_{1}>{\mathcal{S}}_{4}$
q-ROFSWG^44^	− 0.069531	0.045302	0.091285	− 0.057301	${{\mathcal{S}}}_{3}>{{\mathcal{S}}}_{2}>{{\mathcal{S}}}_{4}>{\mathcal{S}}_{1}$
q-ROFSIWA^46^	0.287695	0.393784	0.422568	0.280653	${{\mathcal{S}}}_{3}>{{\mathcal{S}}}_{2}>{{\mathcal{S}}}_{1}>{\mathcal{S}}_{4}$
q-ROFSIWG^46^	0.038572	0.069405	0.097613	0.035632	${{\mathcal{S}}}_{3}>{{\mathcal{S}}}_{2}>{{\mathcal{S}}}_{1}>{\mathcal{S}}_{4}$
q-ROFSEWA^47^	0.409832	0.456041	0.480529	0.364571	${{\mathcal{S}}}_{3}>{{\mathcal{S}}}_{2}>{{\mathcal{S}}}_{1}>{\mathcal{S}}_{4}$
q-ROFSEWG^48^	− 0.176815	− 0.215794	− 0.142093	− 0.157603	${{\mathcal{S}}}_{3}>{{\mathcal{S}}}_{4}>{{\mathcal{S}}}_{1}>{\mathcal{S}}_{2}$
FFSWA^51^	0.764908	0.840453	0.895381	0.785192	${{\mathcal{S}}}_{3}>{{\mathcal{S}}}_{2}>{{\mathcal{S}}}_{4}>{\mathcal{S}}_{1}$
FFSWG^51^	− 0.394613	− 0.374396	− 0.321517	− 0.417152	${{\mathcal{S}}}_{3}>{{\mathcal{S}}}_{2}>{{\mathcal{S}}}_{1}>{\mathcal{S}}_{4}$
q-ROFSAAWA operator	0.018252	0.025915	0.053081	− 0.036378	${{\mathcal{S}}}_{3}>{{\mathcal{S}}}_{2}>{{\mathcal{S}}}_{1}>{\mathcal{S}}_{4}$
q-ROFSAAWG operator	− 0.578591	− 0.613440	− 0.555141	− 0.568486	${{\mathcal{S}}}_{3}>{{\mathcal{S}}}_{4}>{{\mathcal{S}}}_{1}>{\mathcal{S}}_{2}$

These results endorse that the investigation conforms with previous work in articulating an ideal selection, therefore sustaining the integrity and broader implementation of the method proposed to define an FWTT. The development demonstrates the significant upsides and feasibility of the recommended approach. Still, slight discrepancies occur, apparently due to the Aczel-Alsina operators’ capabilities to regulate unclear and conflicting data links while minimizing deformation and information degradation. On the other hand, existing operators noticed challenges in precisely implementing entire and accurate data through their verification protocols.

### Comparison of the proposed operators with different decision-making methods in the same structure

A detailed comparison is executed between the proposed method and different methods in the q-ROFSS structure, with ${{\mathcal{S}}}_{3}$ frequently deemed to be the most effective option for FWTT. The most significant difference consists in this order of alternatives, which arises from the methodologies used to find the positive and negative ideal solutions. Techniques such as TOPSIS^43^ and VIKOR^45^ utilize distinctions between ideal solutions to provide rankings, which produce consequences that can vary from those generated by different techniques. However, the similarity measures^49^ in the q-ROFSS structure evaluate instability by considering the disparity in membership and non-membership data. As obscurity increases, these measures of similarity boost the placement and assessment of choice alternatives, especially in the absence of competing or vague evidence. Our presented methodology, however, centres on score values computed by Aczel–Alcina operators to emphasize the necessity for stabilization among alternatives rather than fixating exclusively on optimal limits. This necessitates a more advanced computational technique, especially when considering the comparative disparities among alternatives.

Table 8 shows the stated Aczel–Alcina operators using q-ROFSNs to boost the reliability of FWTT. In contrast to TOPSIS and VIKOR, which concentrate considerably on determining optimal solutions, this analytical variation highlights the reliability and precision of our strategy. It not only recognizes a suitable option but also presents an improved logical and consistent structure for determining FWTT.

**Table 8:** Comparision of proposed operators with different methods.

	Score values/closeness index				Ranking
TOPSIS method^43^	0.648205	0.612719	0.684162	0.651490	${{\mathcal{S}}}_{3}>{{\mathcal{S}}}_{4}>{{\mathcal{S}}}_{1}>{\mathcal{S}}_{2}$
VIKOR method^45^	0.436127	0.419828	0.478415	0.442535	${{\mathcal{S}}}_{3}>{{\mathcal{S}}}_{4}>{{\mathcal{S}}}_{1}>{\mathcal{S}}_{2}$
Similarity measures^49^	0.518973	0.526964	0.540342	0.534931	${{\mathcal{S}}}_{3}>{{\mathcal{S}}}_{4}>{{\mathcal{S}}}_{2}>{\mathcal{S}}_{1}$
Proposed q-ROFSAAWA	0.018252	0.025915	0.053081	− 0.036378	${{\mathcal{S}}}_{3}>{{\mathcal{S}}}_{2}>{{\mathcal{S}}}_{1}>{\mathcal{S}}_{4}$
Proposed q-ROFSAAWG	− 0.578591	− 0.613440	− 0.555141	− 0.568486	${{\mathcal{S}}}_{3}>{{\mathcal{S}}}_{4}>{{\mathcal{S}}}_{1}>{\mathcal{S}}_{2}$

**Fig. 9:**
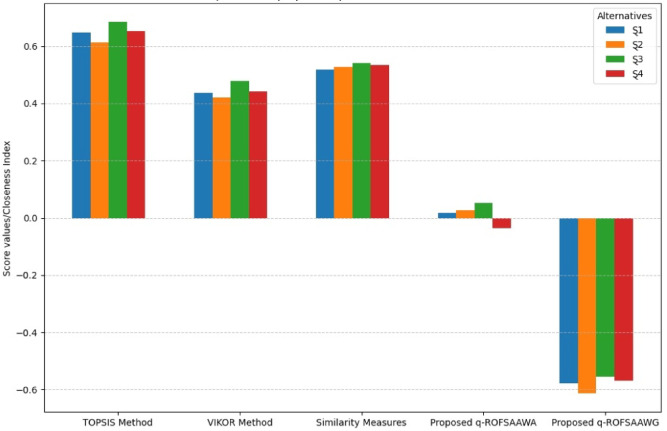
Comparison with different methods.

Figure 9 demonstrates a graphic evaluation of the recommended method and existing approaches. This visual exploration clarified the reliability of our strategy corresponding to different decision-making techniques, presenting a more specific explanation of the inconsistencies in sorting outcomes. This research exposes important distinctions in the estimation of prospects by all techniques. Our proposed method shows a more extensive and detailed outcome by integrating aczel–alsina aggregation operators in the q-ROFSS structure. Figure 9 depicts the more effective efficacy of this approach, especially in managing intricate decision factors and ensuring a uniform exactitude.

Our comprehensive analysis and study confirm that the proposed technique is superior to several existing approaches based on its usefulness in tackling uncertainty and difficulty in decision-making.

### Advantages and theoretical executions of the aczel–alsina-based MAGDM method

The determination of an institution with the most potential to execute an FWTT in this context is complicated. To tackle this concern, we built a detailed framework that includes observations from several directors related to FWTT and the significant challenges they indicate. This process permits a detailed evaluation of the entire business’s strengths and generates an adequate structure for determining the reliability and validity of our behaviour.

The MAGDM approach, which implements Aczel–Alsina operators, constructs an assigned ranking for every prospective option. Throughout the possible outcomes, ${{\mathcal{S}}}_{3}$ occurred as the most optimistic, prevailed by ${{\mathcal{S}}}_{2}$, ${{\mathcal{S}}}_{1}$, and ${{\mathcal{S}}}_{4}$, as determined by the ranking: ${{\mathcal{S}}}_{3}>{{\mathcal{S}}}_{2}>{{\mathcal{S}}}_{1}>{{\mathcal{S}}}_{4}$ with the q-ROFSAAWA operator, and ${{\mathcal{S}}}_{3}>{{\mathcal{S}}}_{4}>{{\mathcal{S}}}_{1}>{{\mathcal{S}}}_{2}$ with the q-ROFSAAWG operator. Most importantly, ${{\mathcal{S}}}_{3}$ continually demonstrates to be a better alternative, resulting in the optimum consequences over multiple scenarios. The ability to adapt facilitates decision-makers to customize attribute weightings to reflect distinctive circumstances, producing a flexible evaluation that satisfies specific criteria.

The improvement of FWM significantly affects the advancement of sustainable development. Evaluation of the organizational effects of FWTT delivers administrators valuable knowledge that may inspire their behavior and determine ordinances that enhance societal growth. Techniques with poor ranks can require more assets and boosted regulation to achieve the efficiency of their superior-ranked competitors.

This methodology is relatively simple to carry out and serves properly when associated with different selection mechanisms. Integrating several experts assures that the precision of the plans and those specified standards or desires deliver noticeable outcomes. Our MAGDM methodology enables the effective determination of the optimal FWTT while tackling realistic problems related to MAGDM in unreliable, realistic situations.

The implementation of dynamic assessment systems provides an improved fundamental appreciation of how variability and separations in intricate networks influence overall actions. In the domain of MAGDM, research conducted^56–58^ about switching mechanisms, fractures, and linear development offers useful knowledge into maintaining convoluted choice structures that require an efficient consolidation of different, inconsistent parameters and opinions from experts.

Moreover, the strength of this approach facilitates FWM and is important to several operational areas. Implementing tools that support decisions and involve legislators in this approach will boost assessment precision and mitigate operational issues. Designing a digital application is helpful to optimize the process of making decisions by maintaining both time and resources. This technique doesn’t just help in resolving issues but also supports reorganizing underutilized resources to boost FWM services.

### Limitations of the proposed aczel–alsina-based MAGDM method

The use of the Aczel–Alsina-based MAGDM strategy in the q-ROFSS context shows several benefits while complementing the average reliability of the proposed method. This interaction facilitates decision-making processes, elevating the performance and reliability of the entire process. Still, it is necessary to appreciate the inherent shortcomings of the methodology. A thorough evaluation of the limitations is imperative for precisely determining the algorithm’s feasibility and determining possible domains for modification.

The interpretations and techniques described in this study can prove context-specific and not necessarily useful across diverse situations, geographical locations, or socioeconomic backdrops. The specifics of this research may affect the relevance and effectiveness of the suggested technique in various environments.

The practicality of this precise MAGDM strategy is strongly based upon its accessibility and integrity of specific information. The differences or imperfections in this information, especially about FWM operations and expert estimations, can affect the reliability of outcomes.

The classification of their priority and ordering of components incorporates expert evaluations, conceivably leading to presumptions. A selection of professional opinions, modified by individual assumptions, may impact the exactitude and dependability of selections.

The method claims that all attributes have a distinct effect on decision-making. In realistic circumstances, aspects commonly communicate, and omitting these interrelations may compromise the overall efficacy of the selection procedure.

## Conclusion

Food waste treatment techniques are continually improving the exchanges among corporations and individuals within their simulated and real-life environments. Even though cloud computing and access are generally accessible, the skills of the basic causes of FWM remain necessary to ensure it promotes evolution for all participants. This research demonstrates imperfections and challenges in FWM and indicates an entire data-driven system to tackle them. Food waste serves as an essential risk to enhance knowledge of the implications of technological breakthroughs in FWM. This study determines the practicality of an FWTT in FWM, consequently confirming its economic merits and presenting a strong foundation for the improvement of administrative mechanisms.

This study evaluates Aczel–Alsina operations for q-ROFSS and defines the q-ROFSAAWA and q-ROFSAAWG operators, showing their basic characteristics. We execute an empirical analysis to assess the best FWTT for food waste management. By conducting comparative and sensitivity studies, we clarify the advantages of the proposed technique over existing methodologies, demonstrating its portability, formulating productivity, and propensity to disclose detailed attribute interaction. These results confirm that the strategy produces thorough, reliable output, rendering it a critical tool for officials, especially in FWM.

Future research can explore the impact of FWTT more broadly on developing policies, company growth, and organizational approaches. The approach employed in this research will be relied on by global food waste administrators to investigate and execute sustainable endeavors, offering an empirical structure for both theoretical and practical evaluations.

## Data Availability

All the data used and analyzed is available in the manuscript.
